# A comprehensive systematic review and meta-analysis of ensifentrine in COPD: dose-dependent effects, safety profile, and GRADE-based certainty of evidence

**DOI:** 10.1007/s00210-025-04558-1

**Published:** 2025-09-11

**Authors:** Amr M. Abou Elezz, Kareem Khalefa, Ahmed Farid Gadelmawla, Lamees Taman, Habiba Tariq Saeed, Amr Alaa Azzouz Elkelany, Habiba Abdelhameed Elrefaey, Mohamed Abo Zeid

**Affiliations:** 1 Faculty of Medicine, Tanta University, Tanta, Egypt; 2 Faculty of Medicine, Menoufia University, Menoufia, Egypt

**Keywords:** Ensifentrine, Ohtuvayre, COPD, FEV1, Meta-analysis

## Abstract

**Abstract:**

This study aims to evaluate the efficacy and safety of ensifentrine in COPD patients. Following the PRISMA guidelines, we conducted a systematic review and meta-analysis, systematically searching PubMed, Web of Science, Scopus, and Cochrane library up to 12 December 2024, for randomized controlled trials (RCTs) evaluating ensifentrine compared to placebo in COPD patients. Eligibility criteria included studies reporting outcomes such as pulmonary function tests, exacerbation rates, and adverse events. Subgroup analysis was conducted based on the timing of outcome evaluation and the doses administered. Additionally, a meta-regression model was employed to evaluate the possible correlations between the Ensifentrine doses and “Average forced expiratory volume (FEV 1)” results and identify the optimal dose. Trial sequential analysis (TSA) was implemented to ensure the conclusiveness of our results. Furthermore, the GRADE approach was used to assess the certainty of evidence and for quality assessment the RoB-2 tool was used. Four RCTs were included in our analysis with a total of 2370 COPD patients. Compared to placebo, ensifentrine 3 mg significantly improved lung functions as measured by change in average FEV1 (MD = 0.09, 95% CI: [0.07 to 0.12]), change in peak FEV1 (0–3 h) (MD = 0.15, 95% CI: [0.13 to 0.18]), and change in morning trough FEV1 (MD = 0.04, 95% CI: [0.02 to 0.07]). Subgrouping based on the administrated dose found that ensifentrine 3 mg showed higher, yet non-significant results compared to the included doses (0.75, 1.5, and 6 mg) in all pulmonary function tests. Moreover, meta-regression revealed a significant dose–response relationship for average FEV1 up to 3 mg, indicating optimal efficacy at 3-mg dose. Ensifentrine also significantly improved quality of life measures, with no significant increase in adverse events across doses. Ensifentrine has proven to be effective in improving lung functions and respiratory symptoms with an acceptable safety profile, thus suggesting a valuable addition to the management of COPD with consideration of potential adverse effects. Nevertheless, further studies with extended long-term follow-up are essential to fully assess the sustained efficacy and safety of ensifentrine and support its optimal therapeutic integration.

**Clinical trial number:**

Not applicable.

**Supplementary Information:**

The online version contains supplementary material available at 10.1007/s00210-025-04558-1.

## Introduction

Chronic obstructive pulmonary disease (COPD) is a syndrome caused by specific pathophysiological processes including innate and adaptive TH1-type immune response to toxins, microbes, or autoimmunity; persistent TH2 inflammation; antiprotease deficiency; and other mechanisms affecting the airways, alveoli, or both causing irreversible airflow obstruction and persistent airway inflammation and symptoms such as dyspnea, cough, and excessive sputum production resulting in a reduced quality of life (QoL) (Devine 2008; Caramori et al. 2016).

Although the current standard of care includes treatment with inhaled short- and long-acting bronchodilators (i.e., long-acting muscarinic antagonists [LAMAs] and long-acting b2-agonists [LABAs]) and inhaled corticosteroids (ICS) which have been shown to improve lung function, COPD symptoms, and health-related QoL and to reduce exacerbation frequency, many patients remain symptomatic and functionally impaired. Furthermore, ICS are associated with a higher risk of pneumonia, including increased risk of hospitalized pneumonia; besides, known cardiovascular and urinary tract risks exist in a subset of patients using LABA and LAMA therapies (Cazzola et al. 1533; Matera et al. 2015, 2020).

There are enzymes known as phosphodiesterase (PDEs) that regulate intracellular levels of cyclic nucleotide signaling molecules to regulate a variety of cellular processes. In airway smooth muscle, PDE3 controls the levels of cyclic adenosine monophosphate (cAMP) and cyclic guanosine monophosphate (cGMP), whereas PDE4 controls the levels of cAMP and has a role in the activation of inflammatory cells. Phosphodiesterase inhibitors (PDEIs) show a promising treatment of obstructive and inflammatory diseases of the respiratory tract, such as COPD, cystic fibrosis, and asthma. It has been demonstrated that dual inhibition of PDE3 and PDE4 has synergistic effects on airway smooth muscle contraction and inflammatory response suppression when compared to inhibition of either PDE3 or PDE4 alone (Boer et al. 1992; Banner and Press 2009).

Ensifentrine offers a novel approach to COPD treatment through its mechanism as a dual PDE3 and PDE4 inhibitor (Faruqi et al. 2024). As a PDE3 inhibitor, ensifentrine raises levels of cAMP in bronchial smooth muscle cells which leads to bronchodilation; therefore, it improves airflow and reduces the feeling of dyspnea in patients with COPD. Ensifentrine also has an anti-inflammatory effect due to its role as a PDE4 inhibitor, which improves lung function and reduces exacerbations in COPD patients (Gan et al. 2024).

The combined effect of ensifentrine makes it superior to other drugs used in COPD treatment. In addition to that, ensifentrine is an inhaled medication allowing for targeted delivery to the lungs with few side effects unlike oral drugs or systemic corticosteroids. Previous trials investigating ensifentrine have encouraged its role in management of COPD, especially at dose of 3 mg which has been recently FDA approved as a nebulized maintenance treatment for COPD (Keam 2024; Anzueto et al. 2023; Ferguson et al. 2021; Singh et al. 2020b).

Previous meta-analyses have evaluated the effects of ensifentrine 3 mg and other doses versus placebo on pulmonary function and adverse events (Hammadeh et al. 2025). However, this study aims to provide a more comprehensive dose–response evaluation by analyzing and comparing multiple doses of ensifentrine using subgroup analyses, implementing trial sequential analysis (TSA), and assessment of the certainty of evidence using the GRADE approach. Additionally, we offer a detailed and meticulous evaluation of all efficacy outcomes across different time points to obtain more rigorous evidence along with an expanded safety analysis to include a broader range of adverse events.

## Methods

### Study design and registration

This systematic review and meta-analysis followed the PRISMA (Page et al. 2021) (Preferred Reporting Items for Systematic Reviews and Meta-Analyses) guidelines and adhered to the Cochrane Collaboration’s recommendations. The GRADE approach was used to assess the strength of the evidence. The protocol for this study was registered in the International Prospective Register of Systematic Reviews (Prospero) under the registration number CRD42024622326.

## Eligibility criteria

In this review, we adopted the PICO framework to establish our inclusion criteria. We included randomized controlled trials (RCTs) that enrolled adults (≥ 18 years) with COPD, emphysema, or chronic bronchitis (Population), comparing ensifentrine at doses of 0.75 to 6 mg (Intervention) with placebo (Comparator), and reporting any efficacy (Average Forced expiratory volume (FEV1), Peak FEV1, Morning Trough FEV1, Weekly Evaluating-Respiratory Symptoms (E-RS) Total Score, St. George’s Respiratory Questionnaire (SGRQ), or Transition Dyspnea Index (TDI) Questionnaire Total Score) or safety outcomes (the incidence of treatment emergent adverse events (TEAEs), Serious TEAEs, COPD attacks, diarrhea, hypertension, or nasopharyngitis).

We excluded studies including patients diagnosed with asthma. As well as non-randomized control trials, RCTs with irrelevant outcomes, or unavailable complete data. Studies included animal experiments, conference reports, reviews, retrospective studies, meta-analyses, and case reports.

## Search strategy

**Fig. 1:**
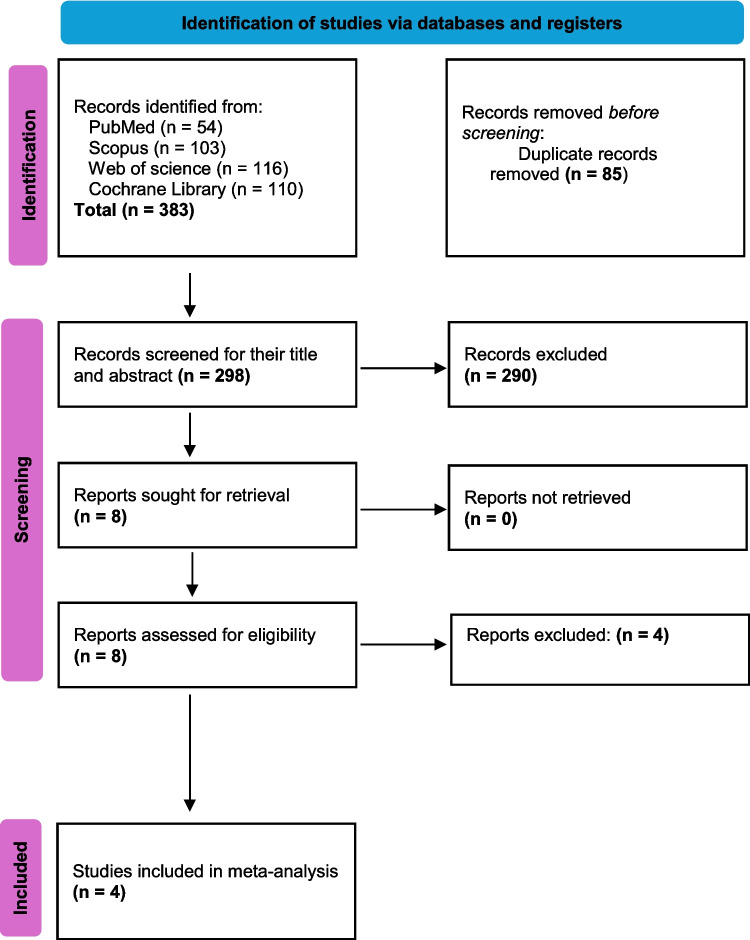
PRISMA Flowchart of included studies

We conducted a systematic search through medical electronic databases: PubMed, Web of Science, and Scopus including studies up to 12 December 2024. The search strategy included Medical Subject Headings (MeSH) targeting studies including the following keywords (“ensifentrine” OR “Phosphodiesterase 3 and 4 Inhibitor” OR “RPL-554” OR “Ohtuvayre”) AND (“Chronic obstructive pulmonary disease” OR “COPD”). (Supplementary Table S1) Search results were then screened through titles and abstracts according to the previously mentioned inclusion and exclusion criteria using Rayyan website (Ouzzani et al., 2016). Then full text screening was done. Screening process is illustrated in the PRISMA flow diagram (Fig. 1).

Two authors screened independently, and any disagreements were resolved with a third author.

## Quality assessment

We assessed the methodological quality of the RCTs by using the Cochrane risk-of-bias tool for randomized trials (RoB-2) (Sterne et al. 2019), which evaluates six key aspects: (1) bias in random sequence generation, (2) bias due to deviations from intended interventions, (3) bias due to missing outcome data, (4) bias in outcome measurement, (5) bias in the selection of reported results, and (6) overall bias. Each aspect is categorized as “low risk” “some concern” or “high risk.” Two authors independently assessed each paper, and any disagreements were resolved with a third author.

## Data extraction

Data from the studies included were extracted into an online data extraction sheet. The extracted data were primarily divided into three main sections: (1) study characteristics, (2) population demographics, and (3) outcome measurements.

Primary outcomes were average, morning through and peak FEV1, SGRQ, TDI questionnaire, and E-RS, while secondary outcomes included all safety outcomes. For FEV₁, a change of approximately ≥ 100 mL is generally regarded as the minimal clinically important difference (MCID) in patients with COPD (Dankers et al. 2020; Donohue 2005). SGRQ evaluates health-related quality of life in patients with airway diseases. It includes three domains, i.e., symptoms, activity, and impact, which covers a range of aspects concerned with social functioning and psychological disturbances resulting from airways disease. Scores range from 0 to 100, with higher scores indicating worse health status. A decrease of ≥ 4 units is regarded as the MID for clinical significance (Jones et al. 1991; Jones and St 2005). TDI measures changes in dyspnea relative to baseline, assessing functional impairment, task magnitude, and effort. TDI scores range from − 9 to + 9, with higher (positive) scores indicating improvement in dyspnea. A change of ≥ 1 unit is typically considered clinically meaningful (Mahler and Witek xxxx). E-RS scale is a patient-reported outcome measure that assesses respiratory symptoms such as cough, sputum, breathlessness, and chest discomfort. It consists of 11 items with a total score ranging from 0 to 40, where higher scores indicate more severe symptoms. A reduction of ≥ 2 units is generally regarded as the MID for clinical significance (Bushnell et al. 2021).

## Data synthesis and statistical analysis

Data analysis was conducted using RevMan Cochrane software (Handbook and for Systematic Reviews of Interventions | Cochrane Training. 2024) (version 5.4). In the absence of heterogeneity, a fixed effect model will be employed, but a random effect model will be utilized in case of heterogeneity.

We analyzed dichotomous data (TEAEs, diarrhea, COPD, nasopharyngitis) using risk ratio (RR), while for continuous data (change from baseline in average FEV1, change from baseline in peak FEV1, change from baseline in morning trough FEV1) we used mean difference (MD) with a 95% confidence interval (CI) and *p*-values < 0.05 were considered statistically significant. All efficacy outcomes were sub-grouped and analyzed on different timepoints (4–6 weeks, 12 weeks, and 24 weeks) to evaluate the cumulative effect of the drug throughout the follow-up periods. Moreover, to ensure consistency in data synthesis, values were standardized to liters (L) from milliliters (mL) prior to analysis. Data was converted to mean and SD using Cochrane’s converting calculator, and in case of missing data a third assessing author was contacted.

## Assessment of heterogeneity

The chi-square test was conducted to evaluate heterogeneity; heterogeneity was considered significant when the chi-square test *p*-value is less than 0.1, in addition to *I*^2^ test used to evaluate heterogeneity according to *I*^2^ classification. *I*^2^ of ≤ 30% is classified as non-significant heterogeneity, 30–50% is classified as moderate heterogeneity, and 70% is classified as significant heterogeneity.

### Meta-regression

A univariate meta-regression analysis was carried out to evaluate the possible associations between the ensifentrine doses (0.75, 1.5, 3, and 6 mg) and the main primary endpoint (average FEV1) results, and identifying the optimal dose, omnibus *p*-value < 0.05 was considered statistically significant, which was preceded by a standard meta-analysis in which the pooled effect sizes along with their 95% confidence intervals were estimated. We conducted the regression meta-analysis using OpenMeta [Analyst] software (by the Center of Evidence Based Medicine, Brown University, School of Public Health, Rhode Island State, USA).

### Trial sequential analysis

To ensure the conclusiveness of our findings and mitigate the risk of false positive results, we implemented a trial sequential analysis (TSA) utilizing the Copen-Hagen TSA program version 0.9.5.10 Beta (Thorlund et al. 2017). For each primary outcome, both monitoring boundaries plot and a Penalized Z-curve Plot were generated ensuring the robustness of our conclusions. We set parameters including a type 1 error (α) of 5% and type 2 error (β) of 20% corresponding to a statistical power of 80%, using a model variance-based heterogeneity correction. As for the Monitoring boundaries plot, we utilized a superiority boundary based on the O’Brien Fleming alpha spending function to mitigate type 1 errors and a futility boundary based on the O’Brien Fleming beta spending function to mitigate type 2 errors. Regarding the Penalized Z-curve Plot, the cumulative *z* statistic was penalized by using the Law of Iterated Logarithm applying a λ value of 2 (Thorlund et al. 2017). Plots included a required information size (RIS) axis to establish if the data is sufficient or not. Empirical data pooled from the included studies were utilized.

## Results

### Search results and study selection

A total of 383 studies were initially identified by using the predefined search strategy on the different databases (PubMed, Scopus, Web of Science, and Cochrane library), 85 of which were duplicated and removed. On screening the titles and abstracts of the remaining 298 studies using the eligibility criteria mentioned above, 290 articles were found irrelevant. When evaluating the full text of the remaining nine articles, only four studies were included in our study (Fig. 1).

### Characteristics of included studies

Four RCTs were included in our analysis with a total of 2370 patients with moderate to severe COPD across different countries (USA, UK, Poland, and Germany). Patients were randomized to receive either different doses of ensifentrine (0.75 1.5, 3, or 6 mg) or placebo, but they were also receiving different maintenance medications like salbutamol, inhaled corticosteroids (ICS), LABA with or without ICS or LAMA, while others did not receive any long-acting maintenance drugs (Table 1). The mean age of participants was ranging between 62.5 years and 65.3 years, and the majority of participants were males 52.42%. About 55.87% of the participants were current smokers with their mean smoking exposure about 44.4 pack per year (Table 2).

**Table 1:** Summary of the included studies

Study ID (Country)	Study design	Study arms		Other drugs taken during the trial	Inclusion criteria	Total participants	Conclusion
		Intervention	Control				
Ferguson 2021(USA)	RCT	Ensifentrine (BID)• 0.375 mg• 0.75 mg• 1.5 mg• 3 mg	Placebo	LAMA	• Symptomatic patients with a pre-dose FEV1 of 30–70%• (mMRC) dyspnea scale score ≥ 2 after 2 weeks of daily treatment with tiotropium	416	Ensifentrine (0.375–3 mg BID) added to tiotropium significantly improved peak FEV1 (78–124 mL) and QoL in symptomatic COPD patients, with a placebo-like safety profile
Singh 2020 (Multi)	RCT	Ensifentrine (BID) • 0.75 mg • 1.5 mg • 3 mg • 6 mg	Placebo	Rescue salbutamol, inhaled corticosteroids	• Males and females aged 40–75 years • Diagnosed with COPD for at least 1 year • Clinically stable for at least 4 weeks • Post-bronchodilator FEV1 between 40 and 80% of predicted normal • FEV1/forced vital capacity (FVC) ratio ≤ 0.7 • Current or former smokers with a smoking history of at least 10 pack-years • Pre-dose FEV1 at randomization within ± 20% of the screening value	405	Ensifentrine significantly improved bronchodilation and symptoms, with a dose-ranging effect from 0.75 to 3 mg twice daily, and all doses well tolerated
Anzueto 2023 (ENHANCE-1) (USA)	RCT	Ensifentrine (BID) • 3 mg	Placebo BID	No long-acting maintenance therapy or LABA with or without ICS or LAMA with or without ICS	• 40–80 years old • Diagnosed with COPD • Post-bronchodilator FEV1 predicted normal between 30 and 70% • FEV1/FVC ratio ≤ 0.7 • mMRC dyspnea scale score > 2 • Smoking history of more than 10 pack-years • No long-acting maintenance therapy or using LABA with or without ICS, or LAMA with or without ICS • Starting or stopping COPD maintenance therapy was not allowed unless medically necessary • Exclusion of patients with asthma	760	Ensifentrine significantly improved lung function in both trials, with results supporting exacerbation rate and risk reduction in a broad COPD population and in addition to other classes of maintenance therapies
Anzueto 2023 (ENHANCE-2) (USA)	RCT	Ensifentrine (BID) • 3 mg	Placebo BID			789	

*LAMA* long-acting muscarinic antagonist, *FEV1* forced expiratory volume in 1 s, *FVC* forced vital capacity, *BID* Bis in Die (Latin), *LABA* long-acting beta-agonist, *ICS* inhaled corticosteroids, *mMRC* Modified Medical Research Council, *QTcF* corrected QT interval (Fridericia's Formula), *DPI* dry powder inhaler, *RCT* randomized controlled trial

**Table 2:** Demographic and baseline characteristics of patients in the included studies

Study ID	Study arms	Study participants	Age (years), mean (SD)	Sex (female), *N* (%)	Race, *N* (%)			post-bronchodilator FEV1		Smoking status, *N* (%)		Mean smoking exposure, pack years (SD)	Maintenance therapy used, *N* (%)			
					White	Black of African American	Others	*L *(SD)	% predicted (SD)	Current	Ex		LAMA	LAMA + ICS	LABA	LABA + ICS
Ferguson 2021	**1.5 mg**	81	63.8 (7.71)	51 (63%)	72 (88.9%)	9 (11.1%)	NA	1.4 (0.38)	49.9 (10.12)	42 (51.9%)	39 (48.1%)	50.5 (25.54)	28 (34.6%)		2 (2.5%)	13 (16.0%)
	**3 mg**	82	64.5 (7.92)	45 (54.9%)	76 (92.7%)	6 (7.3%)	NA	1.4 (0.45)	50.4 (10.61)	43 (52.4%)	39 (47.6%)	51.0 (20.56)	32 (39%)		0	5 (6.1%)
	**Placebo**	84	63.6 (8.41)	44 (52.4%)	75 (89.3%)	9 (10.7%)	NA	1.4 (0.46)	48.9 (10.93)	53 (63.1%)	31 (36.9%)	52.5 (27.37)	43 (51.2%)		2 (2.4%)	13 (15.5%)
Singh 2020	**0.75 mg**	82	63.6 (7.05)	26 (32%)	82 (100%)	NA	NA	1.67 (0.464)	56.0 (10.34)	50 (61%)	32 (39%)	44.7 (21.27)	NA	33 (40%)	NA	NA
	**1.5 mg**	81	63.4 (6.40)	35 (43%)	81 (100%)	NA	NA	1.60 (0.466)	56.0 (9.83)	40 (49%)	41 (51%)	43.7 (21.98)	NA	36 (44%)	NA	NA
	**3 mg**	82	62.5 (6.51)	37 (45%)	82 (100%)	NA	NA	1.62 (0.441)	55.6 (10.18)	47 (57%)	35 (43%)	41.8 (19.05)	NA	29 (35%)	NA	NA
	**6 mg**	80	62.9 (6.73)	32 (40%)	80 (100%)	NA	NA	1.63 (0.474)	55.3 (9.47)	42 (53%)	38 (48%)	37.3 (16.75)	NA	32 (40%)	NA	NA
	**Placebo**	80	63.5 (6.44	30 (38%)	80 (100%)	NA	NA	1.69 (0.493)	56.0 (9.89)	43 (54%)	37 (46%)	43.3 (20.21)	NA	28 (35%)	NA	NA
Anzueto 2023 (ENHANCE-1)	**3 mg**	477	65.1 (7.1)	203 (42.6%)	435 (91.2%)	16 (3.4%)	26 (5.4%)	1.53 (0.46)	52.9 (10.3)	268 (56.2%)	209 (43.8%)	41.1 (20.7)	151 (31.7%)	4 (0.8%)	89 (18.7%)	87 (18.2%)
	**Placebo**	283	64.9 (7.7)	116 (41.0%)	250 (88.3%)	9 (3.2%)	25 (8.3%)	1.51 (0.47)	51.7 (10.5)	163 (57.6%)	120 (42.4%)	41.8 (20.6)	76 (26.9%)	5 (1.8%)	45 (15.9%)	66 (23.3%)
Anzueto 2023 (ENHANCE-2)	**3 mg**	498	65.0 (7.4)	254 (51.0%)	471 (94.6%)	24 (4.8%)	3(0.6%)	1.43 (0.44)	50.8 (10.7)	276 (55.4%)	222 (44.6%)	42.7 (22.9)	168 (33.7%)	1 (0.2%)	34 (6.8%)	72 (14.5%)
	**Placebo**	291	65.3 (7.3)	153 (52.6%)	276 (94.8%)	11 (3.8%)	4(1.3%)	1.42 (0.45)	50.4 (10.7)	160 (55.0%)	131 (45.0%)	41.9 (20.9)	90 (30.9%)	0	23 (7.9%)	47 (16.2%)

*LAMA* long-acting muscarinic antagonist, *LABA* long-acting beta-agonist, *ICS* inhaled corticosteroids

### Quality assessment of the included studies

On using the Cochrane tool for risk of bias 2 on the four included studies, three showed low risk of bias while one demonstrated some concerns mainly in randomization process, missing outcome data, and measurement of the outcomes domains (Supplementary Fig. 1 and 2).

### Efficacy outcomes

#### Pulmonary function assessing outcomes

## Change from baseline in average FEV1 (0–4 h) between ensifentrine 3 mg and placebo

**Fig. 2:**
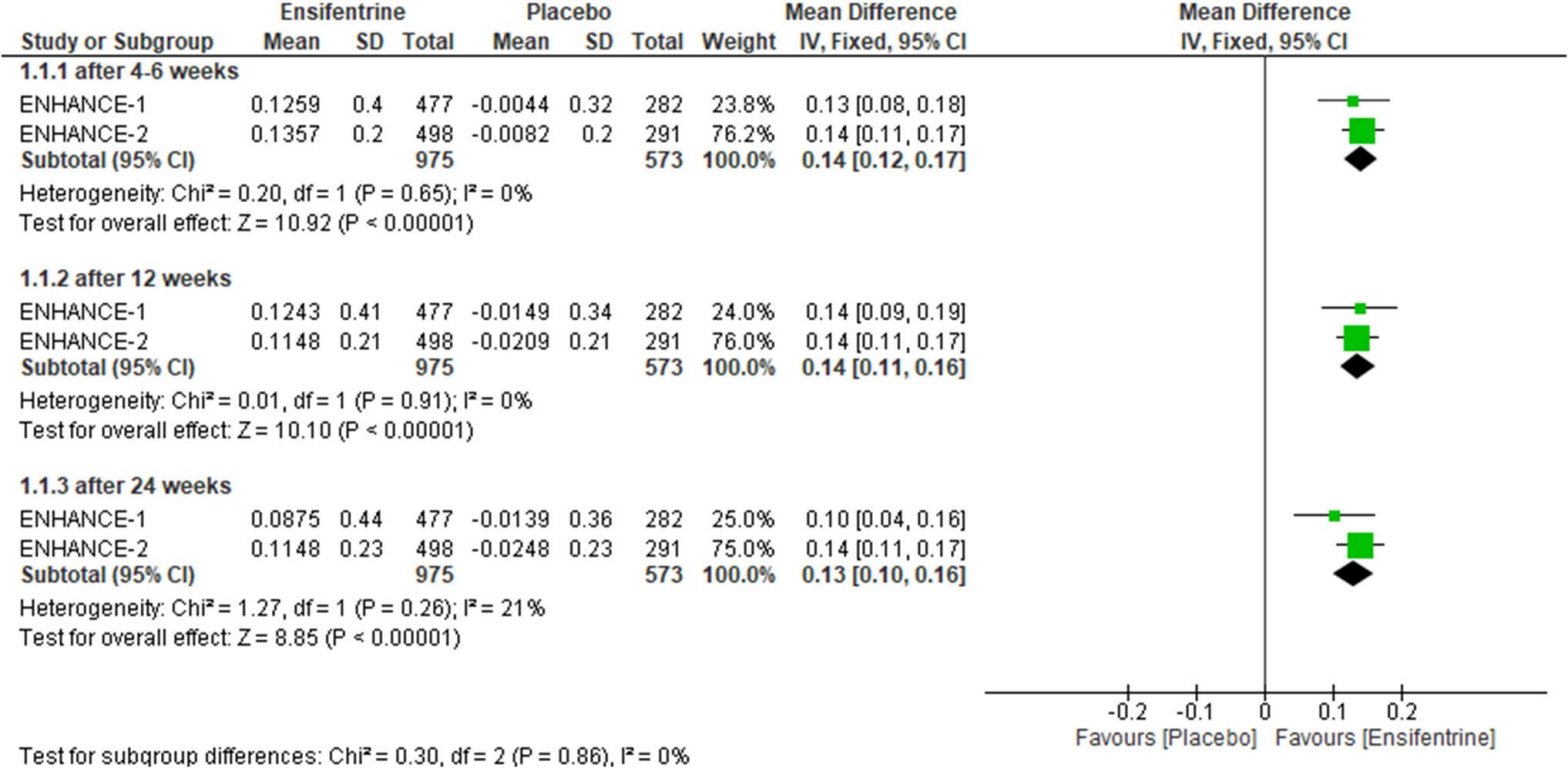
Forest plot comparing (MD) for change from baseline in average FEV1 (0–4 h) between ensifentrine 3 mg and placebo

Change from baseline in average FEV1 was assessed over 4 h in Enchance1,2 trials comparing ensifentrine 3 mg and placebo, and the results were statistically significant favoring ensifentrine in all subgroups, after 4–6 weeks (MD = 0.14, 95% CI: [0.12 to 0.17], *p* < 0.00001), 12 weeks (MD = 0.14, 95% CI: [0.11 to 0.16], *p* < 0.00001), and 24 weeks (MD = 0.13, 95% CI: [0.10 to 0.16], *p* < 0.00001) with no heterogeneity (*I*^2^ = 0%) in first two groups and non-significant heterogeneity in 24 weeks group (*I*^2^ = 21%) and no heterogeneity between subgroups (Fig. 2).

### Change from baseline in average FEV1 (0–12 h) between ensifentrine 3 mg and placebo

**Fig. 3:**
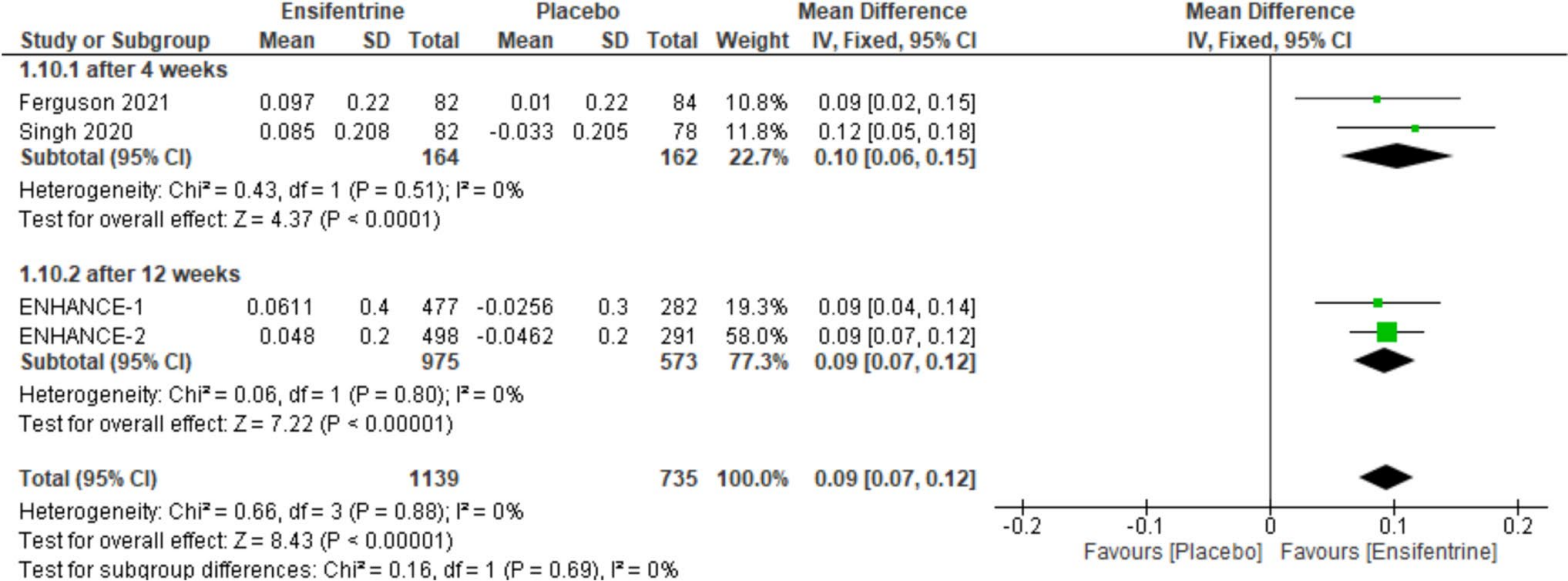
Forest plot comparing (MD) for change from baseline in average FEV1 (0–12 h) between ensifentrine 3 mg and placebo

The same outcome (average FEV1) was assessed over 12 h comparing ensifentrine 3 mg and placebo. The overall effect estimates showed statistically significant difference favoring ensifentrine (MD = 0.09, 95% CI: [0.07 to 0.12], *p* < 0.00001) with no heterogeneity (*I*^2^ = 0%), analyzing subgroups which both showed significant results, after 4 weeks (MD = 0.10, 95% CI: [0.06 to 0.15], *p* < 0.0001), and 12 weeks (MD = 0.09, 95% CI: [0.07 to 0.12], *p* < 0.00001) with no heterogeneity in both subgroups (*I*^2^ = 0%) and no heterogeneity between subgroups (Fig. 3).

The required information size of 233 was reached. Additionally, the final point on the cumulative *z*-curve passed the Superiority boundary (True positive region), indicating a conclusive result (Supplementary Fig. 3A). Moreover, the penalized *Z*-curve passed the conventional boundary of (*z* = 1.96) (Supplementary Fig. 3B).

### Change from baseline in peak FEV1 (between 0 and 3 h) between ensifentrine 3 mg and placebo

**Fig. 4:**
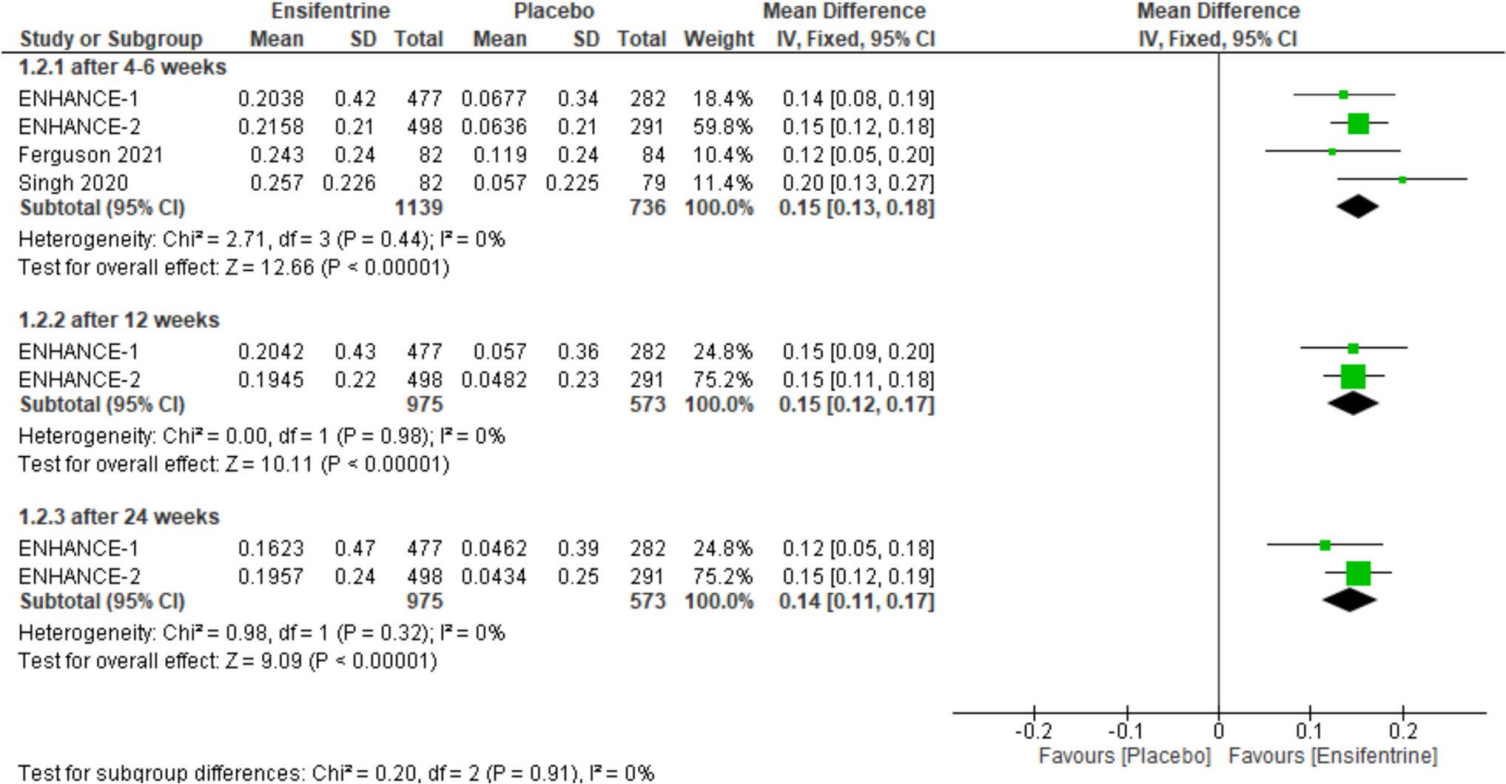
Forest plot comparing (MD) for change from baseline in peak FEV1 (between 0 and 3 h) between ensifentrine 3 mg and placebo

After evaluating change from baseline in peak FEV1 over 3 h to compare ensifentrine 3 mg and placebo, the results were statistically significant favoring ensifentrine in all subgroups, after 4–6 weeks (MD = 0.15, 95% CI: [0.13 to 0.18], *p* < 0.00001), 12 weeks (MD = 0.15, 95% CI: [0.12 to 0.17], *p* < 0.00001), and 24 weeks (MD = 0.14, 95% CI: [0.11 to 0.17], *p* < 0.00001) with no heterogeneity (*I*^2^ = 0%) in all groups and no heterogeneity between subgroups (Fig. 4).

### Change from baseline in morning trough FEV1 between ensifentrine 3 mg and placebo

**Fig. 5:**
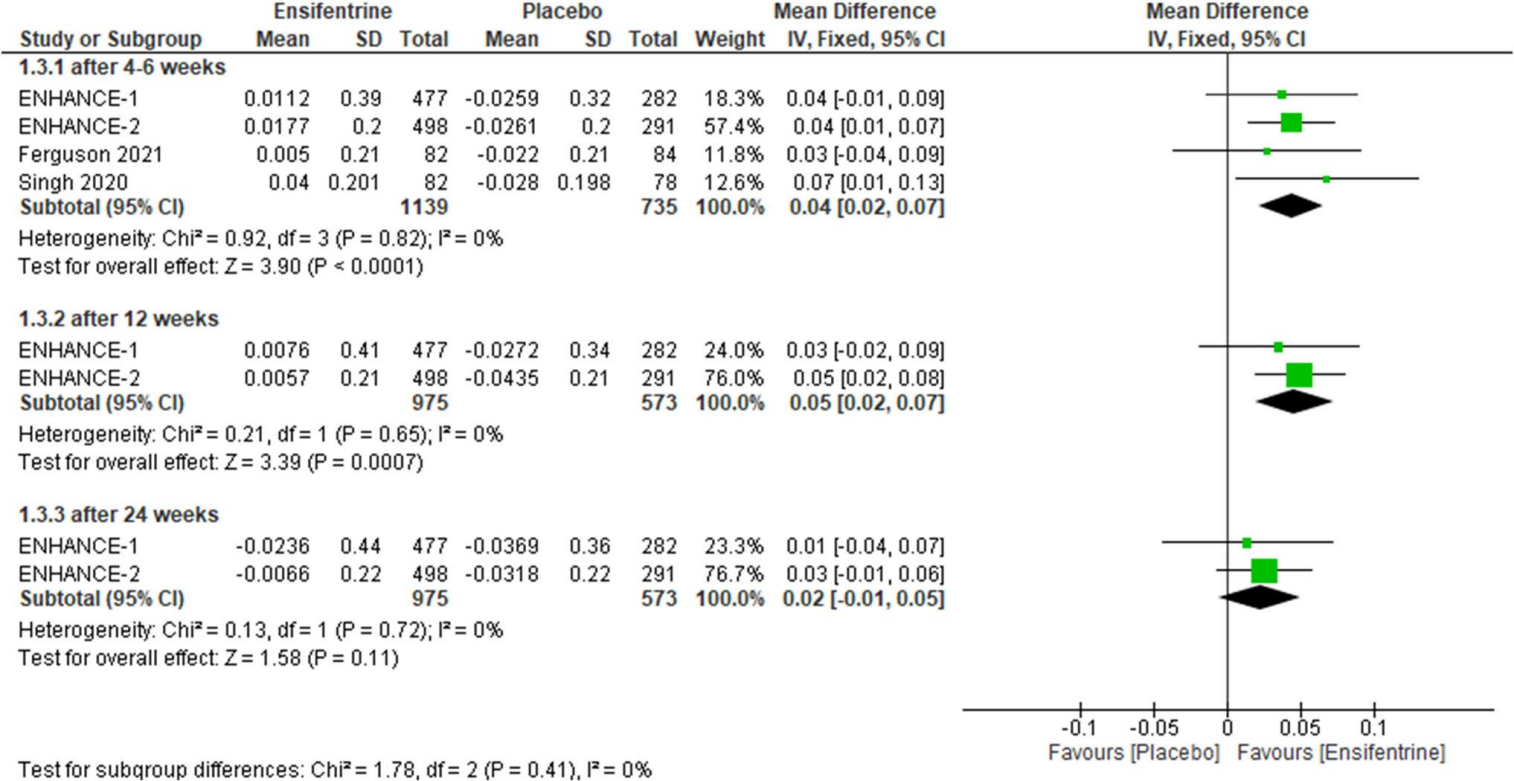
Forest plot comparing (MD) for change from baseline in morning trough FEV1 between ensifentrine 3 mg and placebo

Assessing this outcome to favorable either ensifentrine 3 mg or the placebo, the results were statistically significant favoring ensifentrine after 4–6 weeks (MD = 0.04, 95% CI: [0.02 to 0.07], *p* < 0.0001) and after 12 weeks(MD = 0.05, 95% CI: [0.02 to 0.07], *p* = 0.0007), but after 24 weeks they were not statistically significant (MD = 0.02, 95% CI: [− 0.01 to 0.05], *p* = 0.11) with no heterogeneity (*I*^2^ = 0%) in all groups and no heterogeneity between subgroups (Fig. 5).

The required information size of 1178 was reached. Additionally, the final point on the cumulative *z*-curve passed the Superiority boundary (True positive region), indicating a conclusive result (Supplementary Fig. 4A). Moreover, the penalized *Z*-curve passed the conventional boundary of *z* = 1.96 (Supplementary Fig. 4B).

### Change from baseline in average FEV1 (0–12 h) between ensifentrine 3 mg and 0.75, 1.5, and 6 mg after 4 weeks

**Fig. 6:**
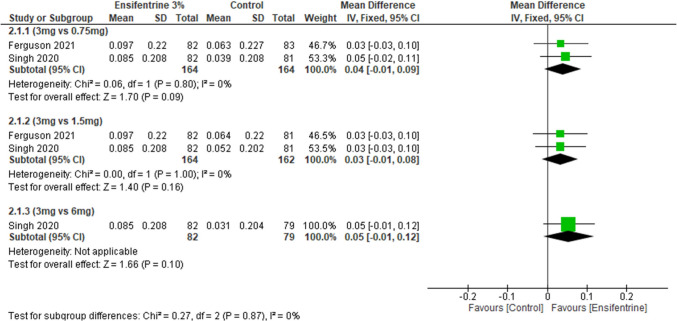
Forest plot comparing (MD) for change from baseline in average FEV1 (0–12 h) between ensifentrine 3 mg and 0.75, 1.5, and 6 mg after 4 weeks

This outcome assessed change from baseline in average FEV1 over 12 h but here it compares ensifentrine 3 mg dose with other doses; it showed statistically insignificant difference between 3 mg dose and other doses, 0.75 mg dose (MD = 0.04, 95% CI: [− 0.01 to 0.09], *p* < 0.09), 1.5 mg (MD = 0.03, 95% CI: [− 0.01 to 0.08], *p* < 0.16), and 6 mg (MD = 0.05, 95% CI: [− 0.01 to 0.12], *p* < 0.10) with no heterogeneity (*I*^2^ = 0%) in all groups and no heterogeneity between subgroups (Fig. 6).

### Change from baseline in peak FEV1 (between 0 and 3 h) between ensifentrine 3 mg and 0.75, 1.5, and 6 mg after 4 weeks

**Fig. 7:**
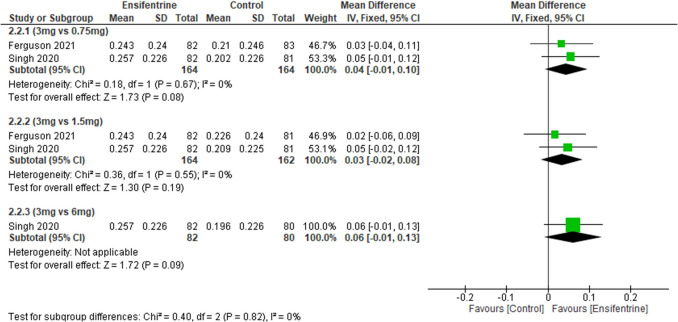
Forest plot comparing (MD) for change from baseline in peak FEV1 (between 0 and 3 h) between ensifentrine 3 mg and 0.75, 1.5, and 6 mg after 4 weeks

Change from baseline in peak FEV1 over 3 h was measured but also here compares ensifentrine 3 mg dose with other doses; results were statistically insignificant to favor any group, 0.75 mg dose (MD = 0.04, 95% CI: [− 0.01 to 0.10], *p* < 0.08), 1.5 mg (MD = 0.03, 95% CI: [− 0.02 to 0.08], *p* < 0.19), and 6 mg (MD = 0.06, 95% CI: [− 0.01 to 0.13], *p* < 0.09) with no heterogeneity (*I*^2^ = 0%) in all groups and no heterogeneity between subgroups (Fig. 7).

### Change from baseline in morning through FEV1 (between 0 and 3 h) between ensifentrine 3 mg and 0.75, 1.5, and 6 mg after 4 weeks

**Fig. 8:**
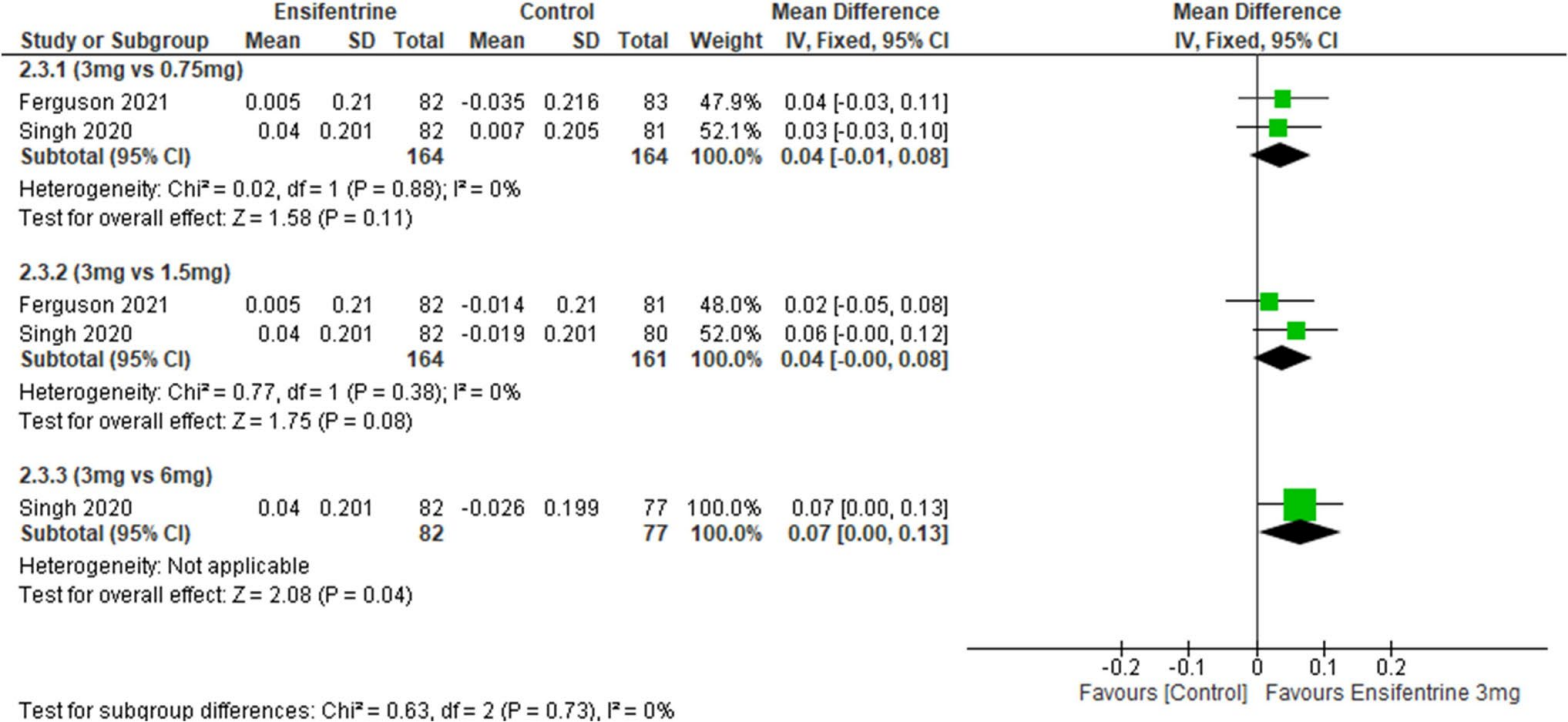
Forest plot comparing (MD) for change from baseline in morning through FEV1 (between 0 and 3 h) between ensifentrine 3 mg and 0.75, 1.5, and 6 mg after 4 weeks

This analysis compares ensifentrine 3-mg dose with other doses; results showed statistically insignificant difference comparing ensifentrine 3-mg dose with 0.75-mg and 1.5-mg doses (MD = 0.04, 95% CI: [− 0.01 to 0.08], *p* < 0.11; MD = 0.04, 95% CI: [− 0.00 to 0.08], *p* < 0.08), respectively, but they were statistically significant favoring 3-mg dose over 6-mg dose (MD = 0.07, 95% CI: [0.00 to 0.13], *p* < 0.04) with no heterogeneity (*I*^2^ = 0%) in all groups and no heterogeneity between subgroups (Fig. 8).

### QoL and symptom improvement

#### Change from baseline in ERS between ensifentrine 3 mg and placebo

**Fig. 9:**
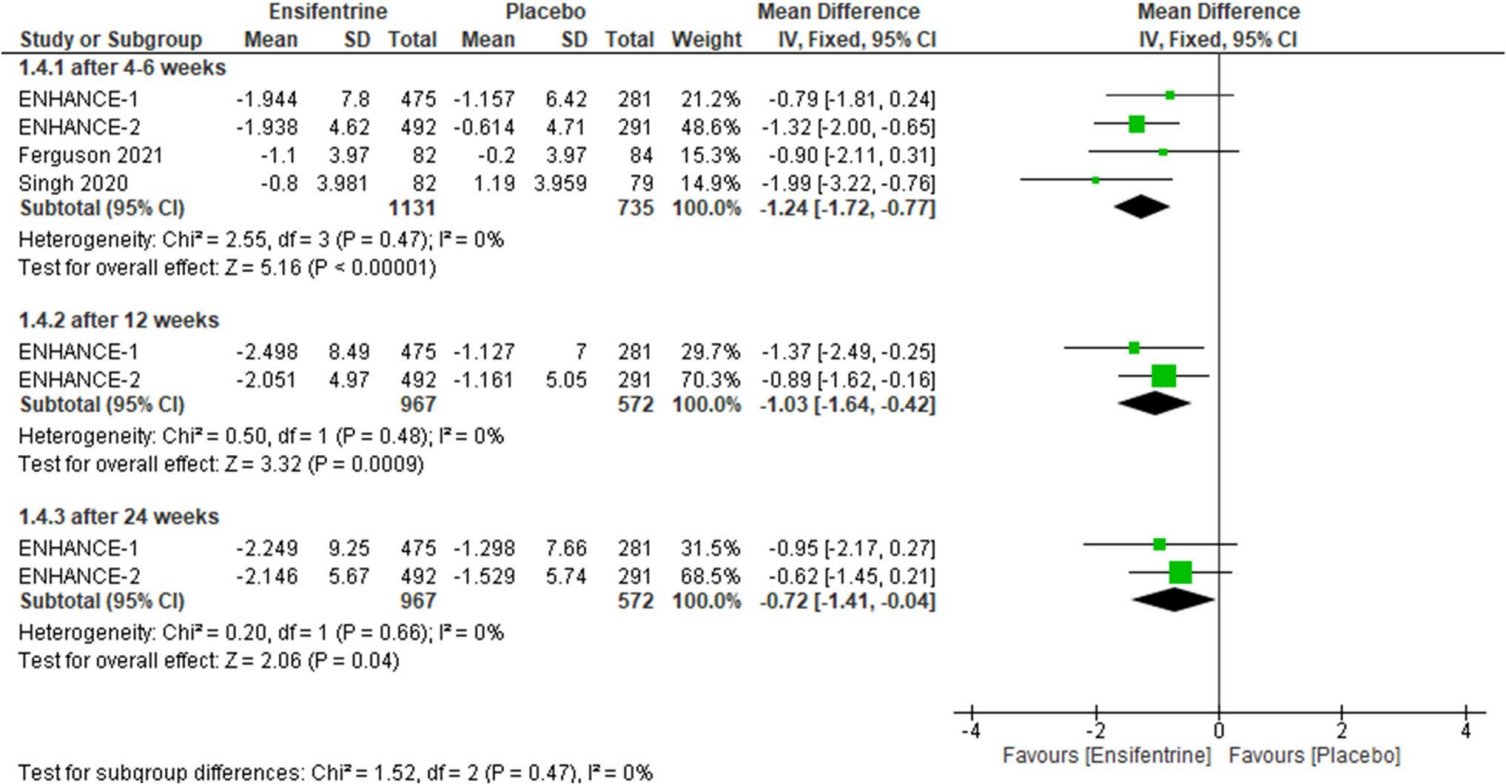
Forest plot comparing (MD) for change from baseline in ERS between ensifentrine 3 mg and placebo

Regarding this outcome, analysis was done to compare ensifentrine 3 mg with placebo and results were statistically significant favoring ensifentrine in all subgroups, after 4–6 weeks (MD =  − 1.24, 95% CI: [− 1.72 to − 0.77], *p* < 0.00001), 12 weeks (MD =  − 1.03, 95% CI: [− 1.64 to − 0.42], *p* = 0.0009), and 24 weeks (MD =  − 0.72, 95% CI: [− 1.41 to − 0.04], *p* = 0.04) with no heterogeneity (*I*^2^ = 0%) in all of them and no heterogeneity between subgroups (Fig. 9).

The required information size of 555 was reached. Additionally, the final point on the cumulative *z*-curve passed the Superiority boundary (True positive region), indicating a conclusive result (Supplementary Fig. 5A). Moreover, the penalized *Z*-curve passed the conventional boundary of *z* = 1.96 (Supplementary Fig. 5B).

#### Change from baseline in SGRQ between ensifentrine 3 mg and placebo

**Fig. 10:**
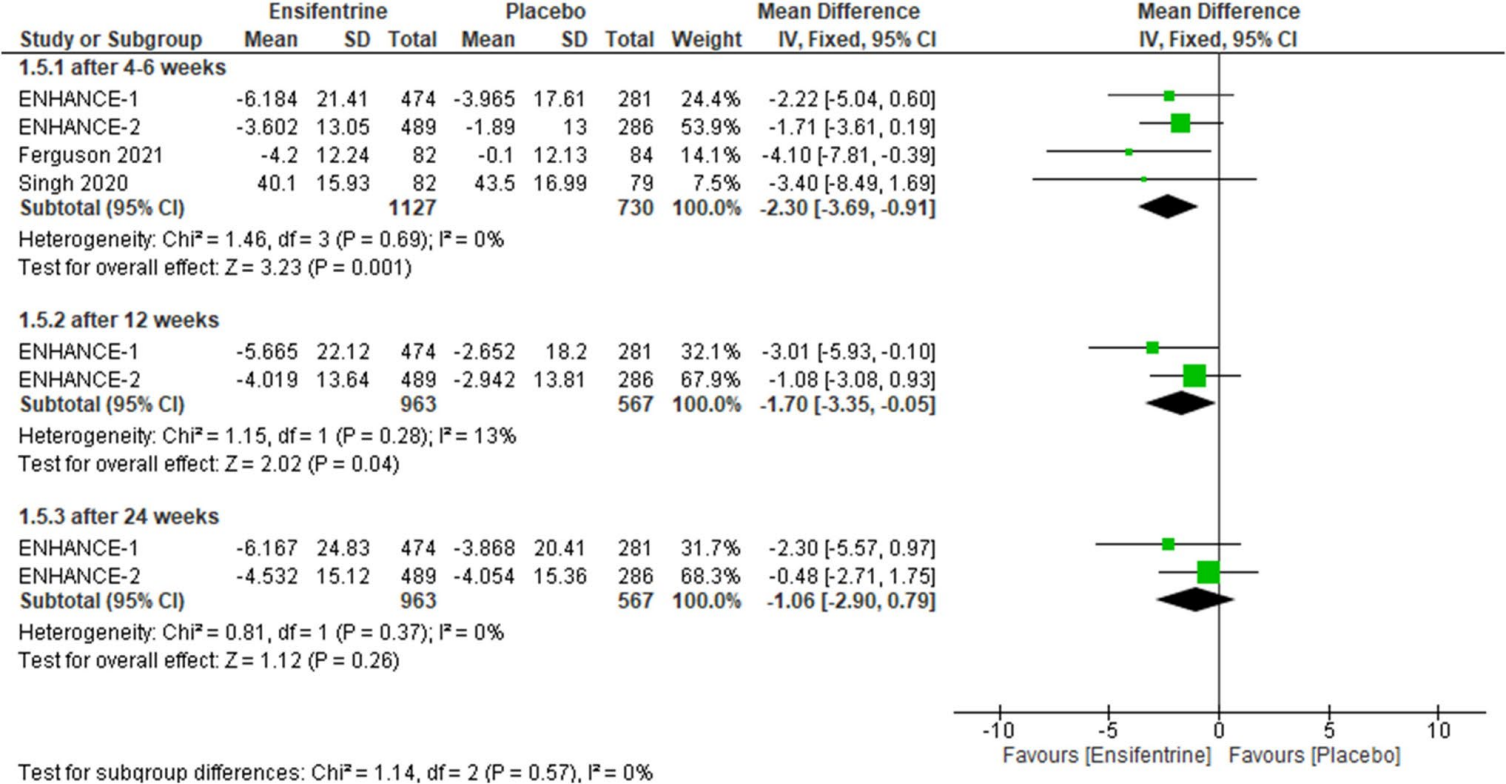
Forest plot comparing (MD) for change from baseline in SGRQ between ensifentrine 3 mg and placebo

As for change from baseline in SGRQ, this outcome contrasted ensifentrine 3 mg with placebo, evaluating revealed statistically significant difference after 4–6 weeks (MD =  − 2.30, 95% CI: [− 3.69 to − 0.91], *p* = 0.001) and after 12 weeks (MD =  − 1.70, 95% CI: [− 3.35 to − 0.05], *p* = 0.04) but after 24 weeks results were statistically insignificant (MD =  − 1.06, 95% CI: [− 2.90 to 0.79], *p* = 0.26), with no heterogeneity (*I*^2^ = 0%) in all groups and no heterogeneity between subgroups (Fig. 10).

The required information size of 1395 was reached. Additionally, the final point on the cumulative *z*-curve passed the Superiority boundary (True positive region), indicating a conclusive result (Supplementary Fig. 6A). Moreover, the penalized *Z*-curve passed the conventional boundary of *z* = 1.96 (Supplementary Fig. 6B).

#### Change from baseline in TDI between ensifentrine 3 mg and placebo

**Fig. 11:**
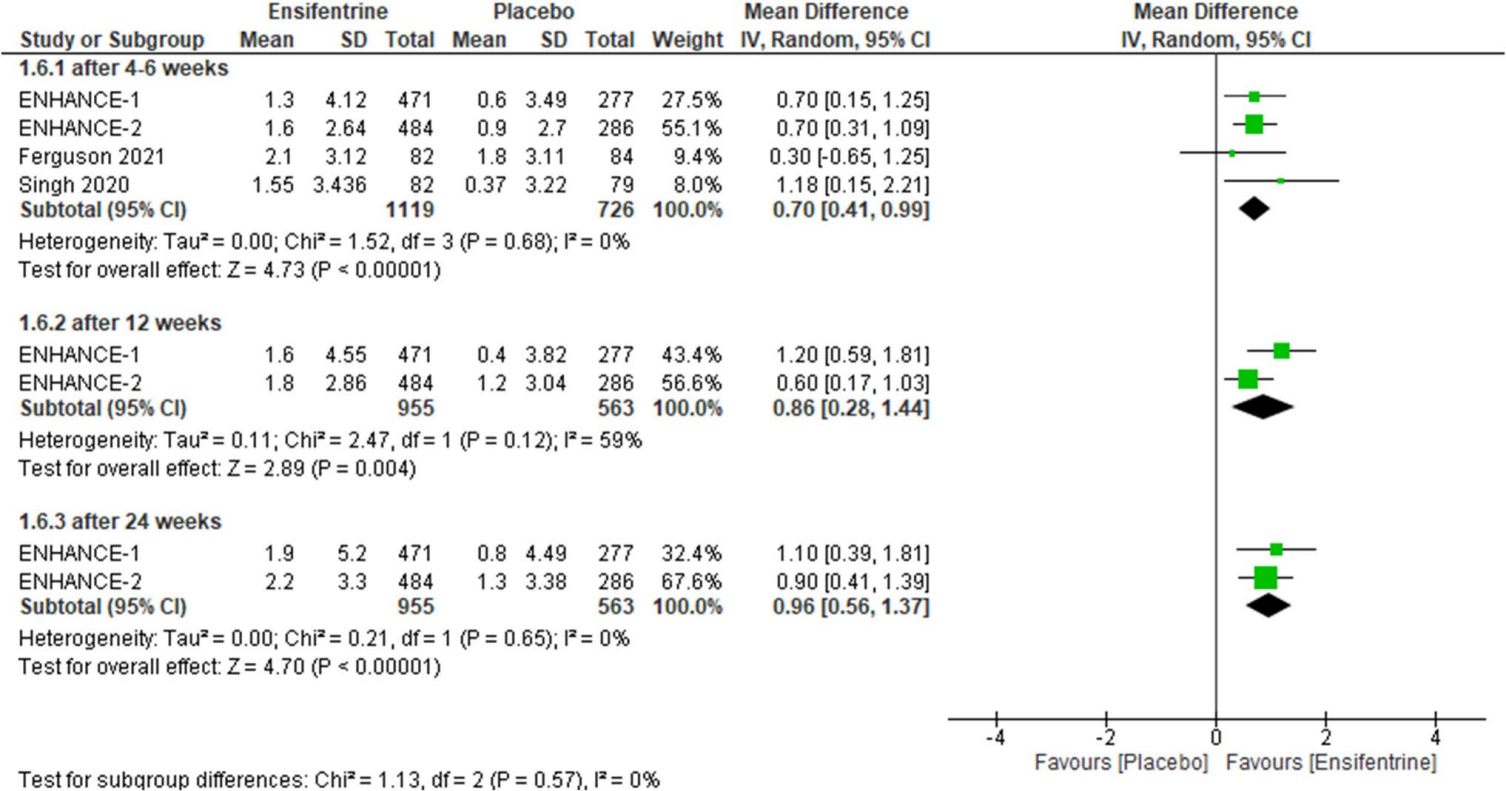
Forest plot comparing (MD) for change from baseline in TDI between ensifentrine 3 mg and placebo

Ensifentrine 3 mg showed statistically significant difference compared to placebo while assessing change from baseline in TDI in all subgroups, after 4–6 weeks (MD = 0.70, 95% CI: [0.41 0.99], *p* < 0.00001), 12 weeks (MD = 0.86, 95% CI: [0.28 to 1.44], *p* = 0.004), and 24 weeks (MD = 0.96, 95% CI: [0.56 to 1.37], *p* < 0.00001) with no heterogeneity (*I*^2^ = 0%) in the first and last groups respectively but there is significant heterogeneity in after 12 weeks group (*p* = 0.12, *I*^2^ = 59%) and no heterogeneity between subgroups (Fig. 11).

The required information size of 1300 was reached. Additionally, the final point on the cumulative *z*-curve passed the Superiority boundary (True positive region), indicating a conclusive result (Supplementary Fig. 7A). Moreover, the penalized *Z*-curve passed the conventional boundary of *z* = 1.96 (Supplementary Fig. 7B).

### Safety outcomes

In *TEAEs*, no statistically significant difference was observed between ensifentrine 3 mg and ensifentrine 0.75, 1.5, and 6 mg or placebo (RR = 1.11, 95% CI [0.78 to 1.58], *p* = 0.56; RR = 0.92, 95% CI [0.67 to 1.28], *p* = 0.64; RR = 0.98, 95% CI [0.65 to 1.47], *p* = 0.91; or RR = 1.02, 95% CI [0.90 to 1.15], *p* = 0.81) (Supplementary Fig. 8). Similarly in number of *COPD attacks*, no statistically significant difference was observed between ensifentrine 3 mg and ensifentrine 0.75, 1.5, and 6 mg or placebo (RR = 0.90, 95% CI [0.32 to 3.05], *p* = 0.98; RR = 1.43, 95% CI [0.13 to 15.72], *p* = 0.64; RR = 0.98, 95% CI [0.20 to 4.69], *p* = 0.98; or RR = 0.90, 95% CI [0.45 to 1.82], *p* = 0.78) (Supplementary Fig. 9). Furthermore, in *nasopharyngitis*, no statistically significant difference was observed between ensifentrine 3 mg and ensifentrine 0.75, 1.5, and 6 mg or placebo (RR = 2.18, 95% CI [0.50 to 9.52], *p* = 0.30; RR = 1.20, 95% CI [0.36 to 4.08], *p* = 0.77; RR = 0.78, 95% CI [0.22 to 2.80], *p* = 0.70; or RR = 0.64, 95% CI [0.38 to 1.09], *p* = 0.10) (Supplementary Fig. 10). As for *Hypertension*, no statistically significant difference was observed between ensifentrine 3 mg and ensifentrine 0.75, 1.5, and 6 mg or placebo (RR = 1.27, 95% CI [0.32 to 5.02], *p* = 0.74; RR = 1.77, 95% CI [0.38 to 8.18], *p* = 0.46; RR = 1.30, 95% CI [0.30 to 5.63], *p* = 0.72; or RR = 2.27, 95% CI [0.93 to 5.54], *p* = 0.07) (Supplementary Fig. 11). Similarly, in *Diarrhea*, no statistically significant difference was observed between ensifentrine 3 mg and ensifentrine 0.75, 1.5, and 6 mg or placebo (RR = 2.32, 95% CI [0.35 to 15.75], *p* = 0.39; RR = 3.93, 95% CI [0.44 to 34.77], *p* = 0.22; RR = 4.88, 95% CI [0.24 to 100.08], *p* = 0.30; or RR = 1.66, 95% CI [0.62 to 4.43], *p* = 0.31) (Supplementary Fig. 12). Finally, as for *Serious TEAEs*, no statistically significant difference was observed between ensifentrine 3 mg and ensifentrine 0.75, 1.5, and 6 mg or placebo (RR = 0.99, 95% CI [0.20 to 4.85], *p* = 0.99; RR = 1.38, 95% CI [0.27 to 6.91], *p* = 0.70; RR = 0.98, 95% CI [0.06 to 15.33], *p* = 0.99; or RR = 1.02, 95% CI [0.69 to 1.51], *p* = 0.90) (Supplementary Fig. 12).

### Meta-regression analysis (dose–response correlation)

**Fig. 12:**
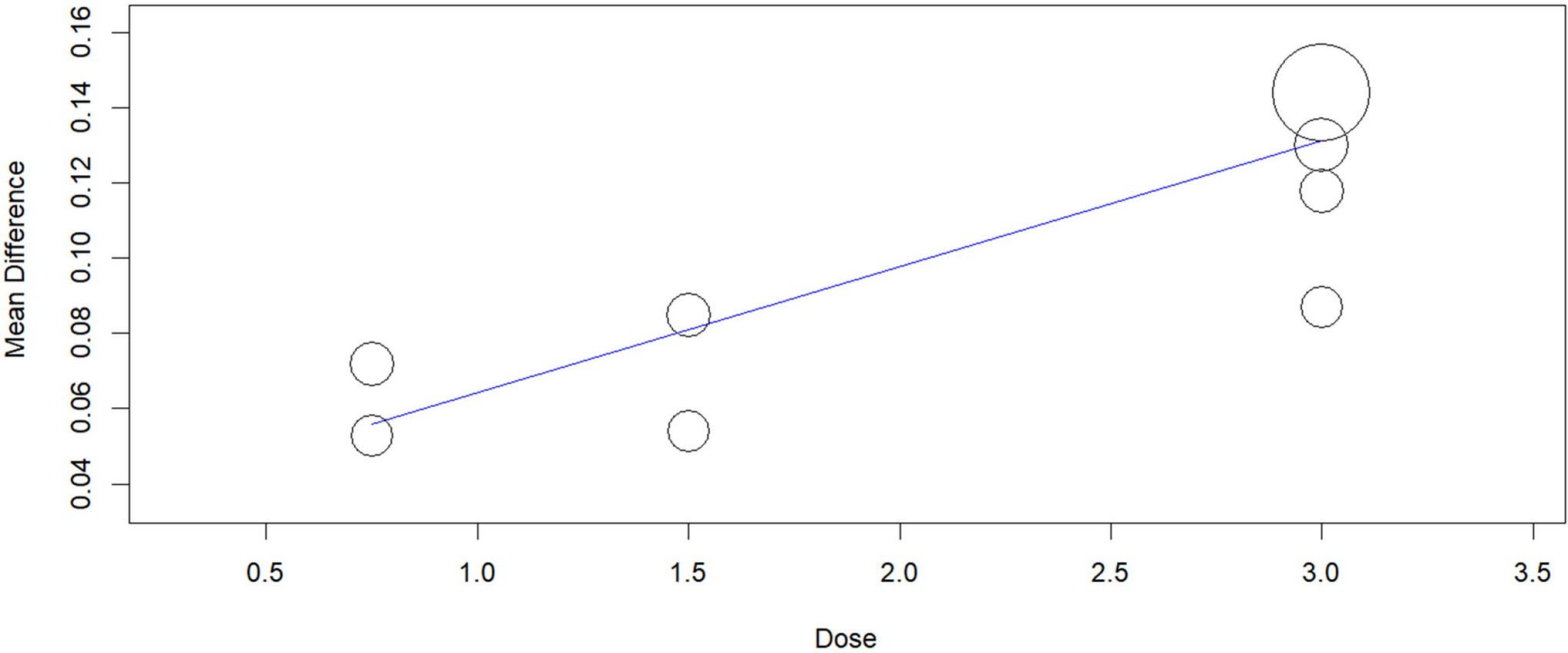
Regression plot for change from baseline in Average FEV1 (excluding 6 mg)

**Fig. 13:**
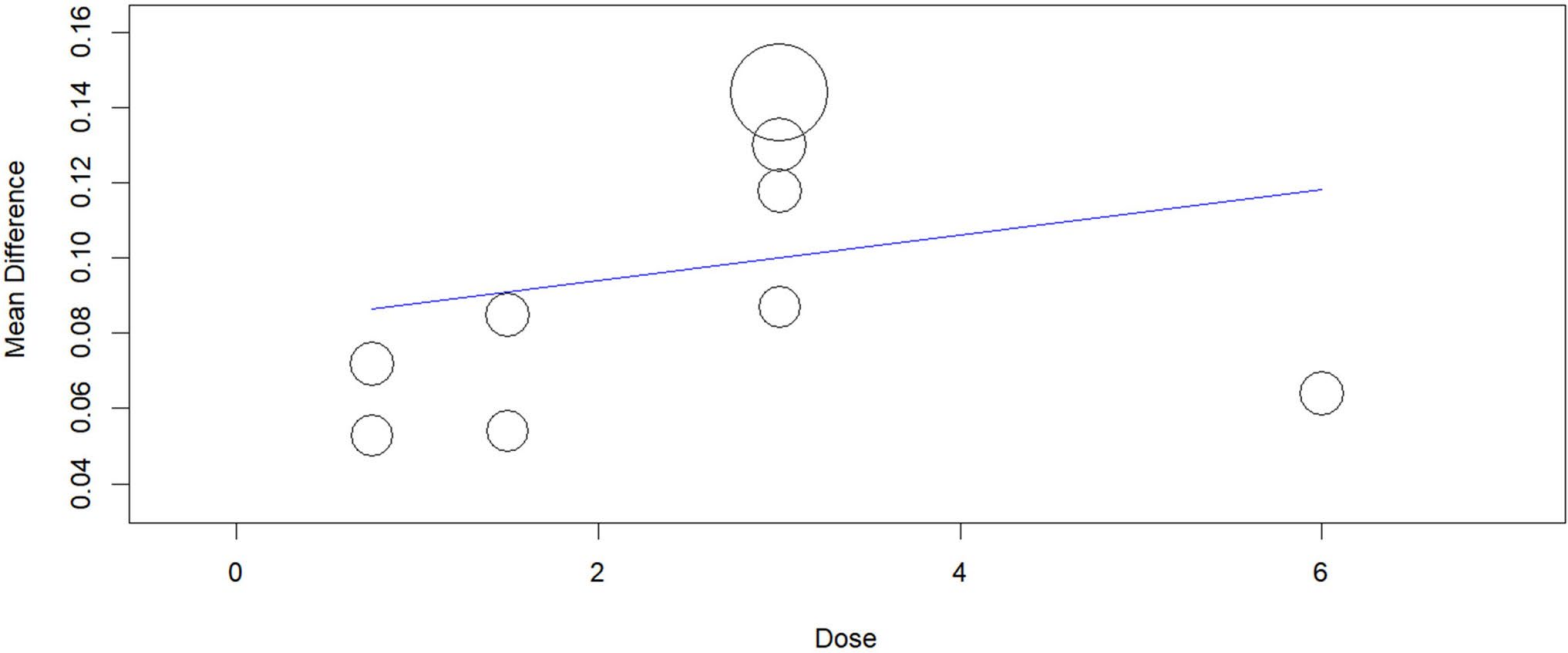
Regression plot for change from baseline in Average FEV1 (including 6 mg)

**Fig. 14:**
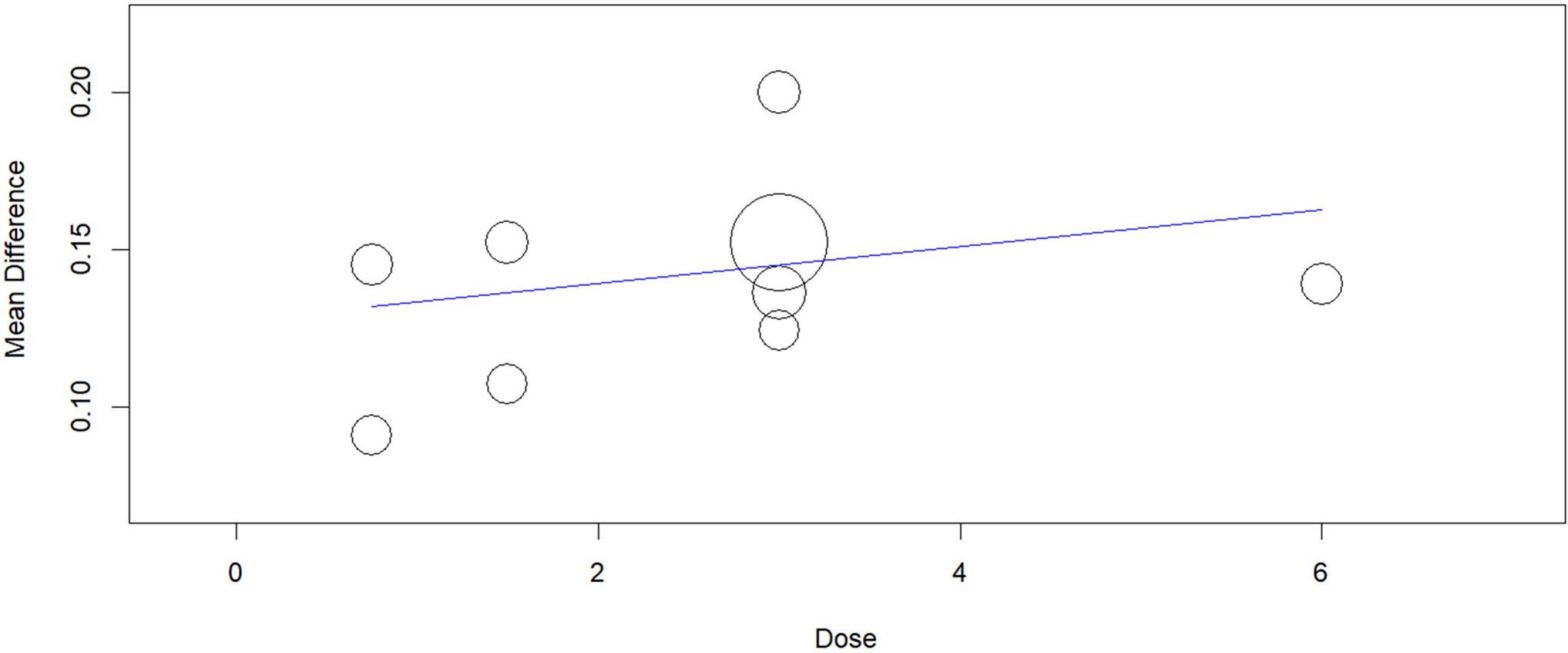
Regression plot for change from baseline in peak FEV1 (including 6 mg)

We conducted a meta regression analysis and found a significant association between the dose of ensifentrine and the effect size at weeks 4–6; the ensifentrine 3-mg dose had the highest benefit over 0.75-mg, 1.5-mg, and 3-mg doses for Average FEV1 (Omnibus *p*-value = 0.001) (Fig. 12). But when adding the 6-mg dose from Singh study, the regression plot showed statistically non-significant results, Average FEV1 (Omnibus *p*-value = 0.483) (Fig. 13). For peak FEV1, results were statistically non-significant (Omnibus *p*-value = 0.445) (Fig. 14).

### Publication bias

Generally, funnel plots revealed a notable degree of symmetry indicating no evidence of publication bias (Supplementary Fig. 14—19).

### GRADE evaluation of evidence

Grade results and summary profile are demonstrated in Table 3. Regarding efficacy outcomes, results mainly demonstrated a high to moderate level of confidence.

**Table 3:** Summary findings and GRADE evaluation of direct comparisons (Ensifentrine vs placebo)

Outcome	Follow up	Dose	No. of studies	No. of patients	MD [± 95% CI]	*p*-value	Heterogeneity assessment *I*^*2*^* [p-value]*	Risk of bias	Indirectness	Inconsistency	Imprecision	Publication bias	GRADE evaluation
Average FEV1 (0–4 h)	4–6 weeks	3 mg	2 RCTs	1548	MD = 0.14, 95% CI: [0.12 to 0.17]	*p* < 0.00001*	*I*^*2*^ = *0% [p* = *0.65]*	No	No	No	No	N/A	**⨁⨁⨁⨁** **High**
	12 weeks	3 mg	2 RCTs	1548	MD = 0.14, 95% CI: [0.11 to 0.16]	*p* < 0.00001*	*I*^*2*^ = *0% [p* = *0.91]*	No	No	No	No	N/A	**⨁⨁⨁⨁** **High**
	24 weeks	3 mg	2 RCTs	1548	MD = 0.13, 95% CI: [0.10 to 0.16]	*p* < 0.00001*	*I*^*2*^ = *21% [p* = *0.26]*	No	No	No	No	N/A	**⨁⨁⨁⨁** **High**
Average FEV1 (0–12 h)	4 weeks	3 mg	2 RCTs	326	MD = 0.10, 95% CI: [0.06 to 0.15]	*p* < 0.0001*	*I*^*2*^ = *0% [p* = *0.51]*	No^a^	No	No	Yes ^b^	N/A	**⨁⨁⨁◯** **Moderate**^**a, b**^
	12 weeks	3 mg	2 RCTs	1548	MD = 0.09, 95% CI: [0.07 to 0.12]	*p* < 0.00001*	*I*^*2*^ = *0% [p* = *0.80]*	No	No	No	Yes ^c^	N/A	**⨁⨁⨁◯** **Moderate**^**c**^
Peak FEV1 (0–3 h)	4–6 weeks	3 mg	4 RCTs	1875	MD = 0.15, 95% CI: [0.13 to 0.18]	*p* < 0.00001*	*I*^*2*^ = *0% [p* = *0.44]*	No ^a^	No	No	No	N/A	**⨁⨁⨁⨁** **High **^**a**^
	12 weeks	3 mg	2 RCTs	1548	MD = 0.15, 95% CI: [0.12 to 0.17]	*p* < 0.00001*	*I*^*2*^ = *0% [p* = *0.98]*	No	No	No	No	N/A	**⨁⨁⨁⨁** **High**
	24 weeks	3 mg	2 RCTs	1548	MD = 0.14, 95% CI: [0.11 to 0.17]	*p* < 0.00001*	*I*^*2*^ = *0% [p* = *0.32]*	No	No	No	No	N/A	**⨁⨁⨁⨁** **High**
Morning trough FEV1	4–6 weeks	3 mg	4 RCTs	1874	MD = 0.04, 95% CI: [0.02 to 0.07]	*p* < 0.0001*	*I*^*2*^ = *0% [p* = *0.82]*	No ^a^	No	No	Yes^d^	N/A	**⨁⨁⨁◯** **Moderate **^**a, d**^
	12 weeks	3 mg	2 RCTs	1548	MD = 0.05, 95% CI: [0.02 to 0.07]	*p* = 0.0007*	*I*^*2*^ = *0% [p* = *0.65]*	No	No	No	Yes^e^	N/A	**⨁⨁⨁◯** **Moderate **^**e**^
	24 weeks	3 mg	2 RCTs	1548	MD = 0.02, 95% CI: [− 0.01 to 0.05]	*p* = 0.11	*I*^*2*^ = *0% [p* = *0.72]*	No	No	No	Yes^e, f^	N/A	**⨁⨁⨁◯** **Moderate**^**e, f**^
ERS	4–6 weeks	3 mg	4 RCTs	1866	MD = − 1.24, 95% CI: [− 1.72 to − 0.77]	*p* < 0.0001*	*I*^*2*^ = *0% [p* = *0.47]*	No ^a^	No	No	Yes ^d^	N/A	**⨁⨁⨁◯** **Moderate **^**a, d**^
	12 weeks	3 mg	2 RCTs	1539	MD = − 1.03, 95% CI: [− 1.64 to − 0.42]	*p* = 0.0009*	*I*^*2*^ = *0% [p* = *0.48]*	No	No	No	Yes ^e^	N/A	**⨁⨁⨁◯** **Moderate**^**e**^
	24 weeks	3 mg	2 RCTs	1539	MD = − 0.72, 95% CI: [− 1.41 to − 0.04]	*p* = 0.04*	*I*^*2*^ = *0% [p* = *0.66]*	No	No	No	Yes ^e, g^	N/A	**⨁⨁⨁◯** **Moderate**^**e, g**^
SGRQ	4–6 weeks	3 mg	4 RCTs	1857	MD = − 2.30, 95% CI: [− 3.69 to − 0.91]	*p* = 0.001*	*I*^*2*^ = *0% [p* = *0.69]*	No ^a^	No	No	Yes ^d^	N/A	**⨁⨁⨁◯** **Moderate **^**a, d**^
	12 weeks	3 mg	2 RCTs	1530	MD = − 1.70, 95% CI: [− 3.35 to − 0.05]	*p* = 0.04*	*I*^*2*^ = *13% [p* = *0.28]*	No	No	No	Yes ^e^	N/A	**⨁⨁⨁◯** **Moderate**^**e**^
	24 weeks	3 mg	2 RCTs	1530	MD = − 1.06, 95% CI: [− 2.90 to 0.79]	*p* = 0.26	*I*^*2*^ = *0% [p* = *0.37]*	No	No	No	Yes ^e^	N/A	**⨁⨁⨁◯** **Moderate**^**e**^
TDI	4–6 weeks	3 mg	4 RCTs	1845	MD = 0.70, 95% CI: [0.41 to 0.99]	*p* < 0.0001*	*I*^*2*^ = *0% [p* = *0.68]*	No ^a^	No	No	Yes^d^	N/A	**⨁⨁⨁◯** **Moderate **^**a, d**^
	12 weeks	3 mg	2 RCTs	1518	MD = 0.86, 95% CI: [0.28 to 1.44]	*p* = 0.004*	*I*^*2*^ = *59% [p* = *0.12]*	No	No	Yes^h^	Yes ^c^	N/A	**⨁⨁◯◯** **Low **^**c, h**^
	24 weeks	3 mg	2 RCTs	1518	MD = 0.96, 95% CI: [0.56 to 1.37]	*p* < 0.0001*	*I*^*2*^ = *0% [p* = *0.65]*	No	No	No	Yes ^c^	N/A	**⨁⨁⨁◯** **Moderate **^**c**^

*CI* confidence interval, *MD* mean difference

**GRADE Working Group grades of evidence**

**High certainty:** we are very confident that the true effect lies close to that of the estimate of the effect

**Moderate certainty:** we are moderately confident in the effect estimate: the true effect is likely to be close to the estimate of the effect, but there is a possibility that it is substantially different

**Low certainty:** our confidence in the effect estimate is limited: the true effect may be substantially different from the estimate of the effect

**Very low certainty:** we have very little confidence in the effect estimate: the true effect is likely to be substantially different from the estimate of effect

N/A = Although funnel plots revealed a notable degree of symmetry, Egger’s test is not applicable because of the small number of included studies

^a^Some concerns in randomization process, missing outcome data and measurement of the outcomes domains but not rated down to risk of bias

^b^Although TSA revealed conclusive results with a sample size exceeding the RIS, Imprecision was downgraded owing to the wide 95% CI which includes the MCID

^c^Downgraded for imprecision owing to the wide 95% CI which includes MCID

^d^Although TSA revealed conclusive results with a sample size exceeding the RIS, Imprecision was downgraded owing to the pooled 95% CI which is below the MCID

^e^Downgraded for imprecision owing to 95% CI which is below the MCID

^f^Downgraded for imprecision owing to the wide 95% CI which includes the null “0”

^g^Downgraded one level for borderline effect (CI close to null, *p* near 0.05)

^h^Downgraded one level for inconsistency (*I*^2^ > 50%, moderate heterogeneity)

## Discussion

This systematic review and meta-analysis included four RCTs comprising a pooled population of 2370 patients with moderate-to-severe COPD. Our analysis reported that ensifentrine 3 mg demonstrated a statistically and clinically significant difference compared to placebo in improving the pulmonary function of COPD participants, as indicated by the improvement in the variable forms of FEV1 scores reported in our analysis. The improvement was dose-dependent, with a similar maximum efficacy reached at 3- and 6-mg doses. Furthermore, ensifentrine was associated with a significant improvement in the symptom burden and Quality of Life (QoL) of COPD patients compared to placebo, as evidenced by the improvement in the TDI, SGRQ, and E-RS scores, while demonstrating an excellent safety profile compared to placebo. Overall, the reported efficacy and safety of ensifentrine make it a potentially viable option to be included in the therapeutic regimen of COPD, especially as an adjuvant treatment for inadequately controlled COPD patients on LAMA or LABA. However, the limitations present in the data from the included studies weaken the overall evidence, highlighting the need for careful interpretation of our review.

Our analysis showed that ensifentrine at a dose of 3 mg was superior to placebo in improving the respiratory function and reducing the resistance and obstruction of the airways evident by the statistically significant improvement demonstrated in the FEV1 score when assessed over 4 h and 12 h after administration of ensifentrine; this improvement was maintained throughout the entire follow-up period of the included studies that ranged from 4 to 24 weeks. The positive effect of ensifentrine on the FEV1 score was consistent with other findings reported in preliminary studies and meta-analyses (Hammadeh et al. 2025; Singh et al. 2018; Franciosi et al. 2013; Bjermer et al. 2019). Phosphodiesterases (PDEs) are a family of enzymes that regulate many physiological processes in the body by interacting with intracellular second messenger cyclic nucleotides, such as cAMP and cGMP. PDE3 is mainly found in the smooth muscles of blood vessels and different parts of the airway tract, maintaining the muscle tone of these structures, whereas PDE4 is the major enzyme responsible for degrading cAMP in leukocytes, leading to an increase in the production of pro-inflammatory proteins, resulting in the sustaining and spreading of inflammation (Rabe et al. 2023; MacLeod et al. 2021). It was hypothesized that inhibition of PDE3 or PDE4 could have a role in the treatment of COPD, as inhibition of PDE3 could decrease the muscular tone of the airway tracts, leading to bronchodilatation and reduction of airway obstruction, while inhibition of PDE4 could lead to inhibition of the inflammatory process, which is considered to be one of the main pillars of COPD pathology (Kahnert et al. 2023; Vogelmeier et al. 2020). Trials on PDE4 inhibitors have reported that the anti-inflammatory effects of these drugs have proven to be effective in improving the clinical condition of patients with COPD (Rennard et al. 2011; Singh et al. 2020a). The dual mechanism of action of ensifentrine enables it to inhibit both PDE3 and PDE4 at the same time compared to other conventional drugs (Keam 2024). Dual inhibition of PDE3 and PDE4 have already been proven in the literature to have a synergistic effect and being more effective than using either one of the two alone (Zuo et al. 2019). In summary, the improvement in the average FEV1 from the baseline reported in our analysis could be attributed to the bronchodilator and anti-inflammatory effects of ensifentrine.

Ensifentrine 3 mg demonstrated a statistically significant improvement in peak FEV₁ within 3 h post-treatment, indicating its rapid onset of action and potential value in managing acute COPD exacerbations. These results align with those from previously published early-phase clinical trials and may be attributed to a reduction in lung hyperinflation, which is commonly observed early in treatment (Calzetta et al. 2013; Turner et al. 2016; O’Donnell et al. 2011). This improvement was sustained over 24 weeks in both ENHANCE trials suggesting that ensifentrine could have the ability to maintain its efficacy over longer periods of use. The long-term efficacy of ensifentrine is further supported by the improvement in change from baseline in morning through FEV1 in the experimental group compared to the placebo which was statistically significant after 12 weeks and clinically significant after 24 weeks.

We observed that the effect of ensifentrine on FEV1 was dose-dependent, with a maximum clinically meaningful effect at a dose of 3 mg. Our meta-regression analysis showed that ensifentrine at the 3-mg dose had a higher benefit on average FEV1 than 0.75-mg, 1.5-mg, and 6-mg doses; however, this dose effect response correlation was not significant in Peak FEV1.

COPD is an incurable chronic disease that causes heavy mental and physical distress in patients, leading to a reduced quality of life (QoL) (Jacques et al. 2024). TDI is a tool used to assess the severity of dyspnea in COPD patients and the magnitude of its effect on the functional impairment it inflicts upon the patients (Mahler and Witek 2005), while the SGRQ is another validated questionnaire used to measure the health-related quality of life of COPD patients based on their presenting symptoms and the effect on their personal life (Swigris et al. 2017; Buhl et al. 2024; Jones and George’ S. St. 2005). Our analysis reported that ensifentrine 3 mg caused a significant improvement in both the TDI and SGRQ scores compared to placebo, indicating the value of ensifentrine in improving the QoL of patients with COPD. Ensifentrine was also superior to placebo in improving the E-RS score, implying that its positive effect on the QoL is driven by its ability to reduce the symptomatic burden of patients. Watz et al. reported that ensifentrine was superior to placebo in enhancing the symptomatic profile of COPD patients, as demonstrated by a statistically significant improvement in QoL scores, consistent with our finding (Watz et al. 2020).

Regarding the safety analysis, ensifentrine demonstrated an excellent safety profile, and our results did not report any statistically significant difference between ensifentrine 3 mg and placebo in any section of our safety analysis. Furthermore, there was no statistically significant difference between the 3-mg dose and any other dose, further supporting the theory that 3 mg is the optimal dose. Ensifentrine’s safety profile is more favorable than that of oral PDE4 inhibitors, which are associated with a high risk of gastrointestinal side effects, and ICS, which carry an elevated risk of oral and pulmonary infections (Wright et al. 2024; Hubert et al. 2024). Additionally, both ENHANCE trials reported a high incidence of participant withdrawal, but the majority was due to the COVID-19 pandemic, and withdrawal due to TEAE was low and similar between the placebo and experimental groups, while Ferguson et al. reported a very high compliance rate in both the intervention and placebo groups (Anzueto et al. 2023; Ferguson et al. 2021). The pooled analysis of both ENHANCE trials reported a 40% reduction in the rate of COPD exacerbation in the ensifentrine group compared to the placebo group over a period of 24 weeks, and that reduction was still sustained through the subgroup of ENHANCE-1 that was followed up until 48 weeks (Mahler et al. 2024). Thus, we can conclude that ensifentrine could be an effective and safe add-on therapeutic to LABA or LAMA in the treatment of complicated COPD exacerbation as an alternative to the high-risk side-effect-prone ICS.

Our findings build upon those reported by Hammadeh et al. (2025) (Hammadeh et al. 2025), as they conducted a similar meta-analysis on the efficacy and safety of ensifentrine in COPD. Although both studies reported significant improvements in respiratory symptoms scores as well as quality of life (Qol) and symptom improvement our analysis differs in main aspects, we conducted a GRADE evaluation allowing our findings to be interpreted within context of clinical certainty and providing clarity for clinical application. Moreover, while Hammadeh et al. (2025) (Hammadeh et al. 2025) performed dosage subgroup analysis, our study employed comparisons of different doses in addition to the meta-regression analysis to statistically assess dose–response relationships, which revealed the significant association between dose and FEV1 improvement up to 3 mg as the optimal dose.

Additionally, we expanded our safety analysis including specific adverse outcomes such as diarrhea, nasopharyngitis, hypertension, and serious TEAEs, providing a more comprehensive view of ensifentrine’s safety profile. Moreover, our analysis included a subgroup analysis stratified by follow-up duration (4, 12, and 24 weeks). Cumulatively, our methodological additions give a more detailed synthesis of the available evidence.

We conducted this systematic review and meta-analysis to investigate the efficacy and safety of ensifentrine compared to placebo in the treatment of COPD. We included multiple doses, conducted dose vs dose analysis, and analyzed multiple outcomes over different time points. Despite the overall good performance of ensifentrine compared to placebo, our results should be interpreted with caution because of the limited number of clinical trials included and the high withdrawal rate observed in the ENHANCE trials, which could have affected the confounding factors of the included population. Furthermore, the follow-up of the included RCTs ranged from 4 to 24 weeks, which is not adequate to judge the durability of drug effectiveness over a long period of time. The included populations had variable but limited background medications, as many of them did not receive any background treatment, and none were on the newly guideline-including combination of LABA and LAMA, limiting our ability to generalize the effect of adding ensifentrine to other COPD treatment combinations. Thus, in order to fully evaluate the real efficacy and safety of ensifentrine and its value in the current clinical practice, we recommend conduction of further long-term, large-sized, and well-established RCTs that comprise a large number of COPD patients on different forms of COPD drug combinations, especially the combination of LABA and LAMA. We also recommend the conducting RCTs that directly compare ensifentrine to ICS in the treatment of COPD patients, especially as an add-on treatment in COPD exacerbation to fully understand the place of ensifentrine and other PDEI drugs in the current regimens of COPD therapies.

## Conclusion

Finally, our meta-analysis demonstrated the ability of ensifentrine in improving the clinical conditions of COPD patients, as evidenced by the significant change in the pulmonary function test, QoL scores, and favorable safety profile, supported by our TSA findings indicating the conclusiveness of our results. Our findings support ensifentrine potential inclusion as an adjuvant treatment for COPD patients resistant to standard treatment. However, caution is advised when interpreting our results due to the limitations of the included trials. Therefore, further studies with extended long-term follow-up are essential to fully assess the sustained efficacy and safety of Ensifentrine and support its optimal therapeutic integration.

## Supplementary Information

Below is the link to the electronic supplementary material.

ESM 1 (1.79 MB DOCX)

## Data Availability

The datasets used and/or analyzed during the current study are available from the corresponding author on reasonable request.

## Abbreviations

COPD: Chronic obstructive pulmonary disease
FEV1: Forced expiratory volume in 1 s
ERS: Evaluating respiratory symptoms
SGRQ: St. George’s Respiratory Questionnaire
TDI: Transition dyspnea index
RCT: Randomized controlled trial
ICS: Inhaled corticosteroids
LABA: Long-acting beta-agonist
LAMA: Long-acting muscarinic antagonist
PDE: Phosphodiesterase
PDEI: Phosphodiesterase inhibitor
cAMP: Cyclic adenosine monophosphate
cGMP: Cyclic guanosine monophosphate
TEAE: Treatment-emergent adverse event
QoL: Quality of life
mMRC: Modified Medical Research Council
FVC: Forced vital capacity
RoB-2: Risk of Bias tool for randomized trials
