# Supplementary material for: A comprehensive systematic review and meta-analysis of ensifentrine in COPD: dose-dependent effects, safety profile, and GRADE-based certainty of evidence
Source: Naunyn Schmiedebergs Arch Pharmacol. 2025 Sep 11;399(2):2797–818. doi: 10.1007/s00210-025-04558-1 (PMC12901268; doi:10.1007/s00210-025-04558-1)
Supplement: Supplementary file 1 — (1.79 MB DOCX) [file 210_2025_4558_MOESM1_ESM.docx]

**
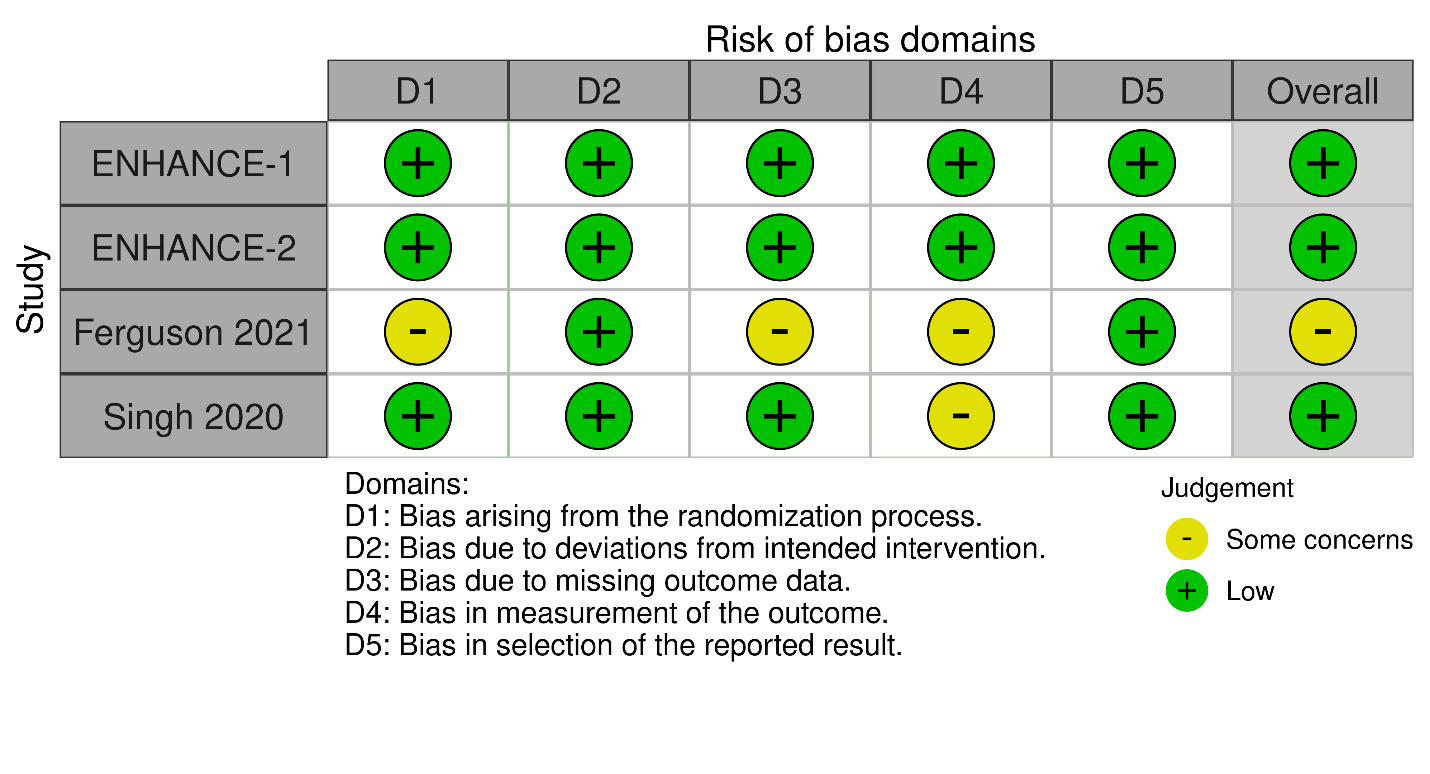
**

**(Supplementary figure. 1)** The bias-risk assessment diagram of the included articles


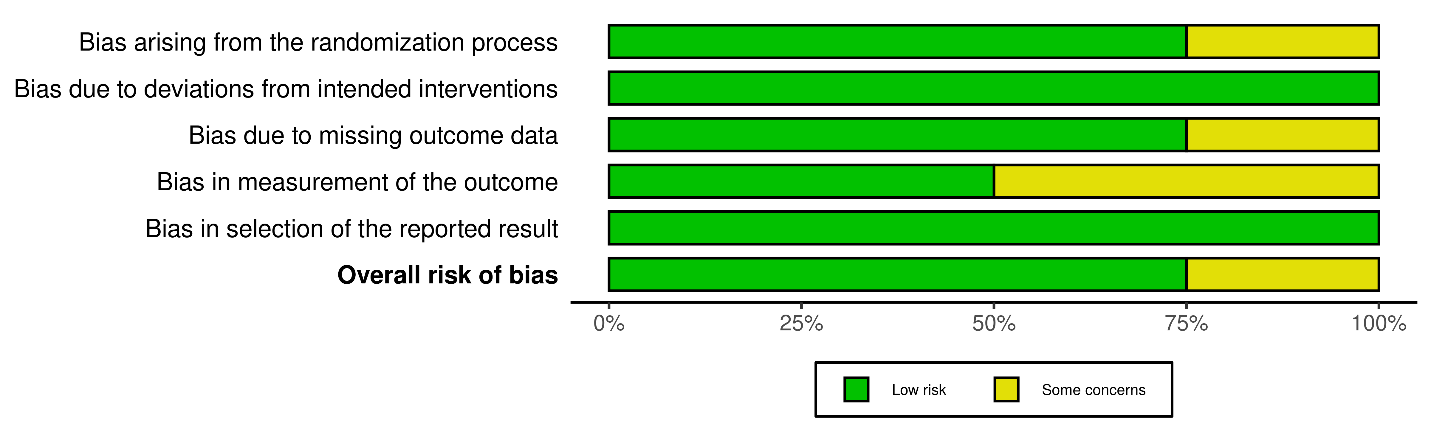


**(Supplementary figure. 2)** The bias evaluation bar graph of the included articles.


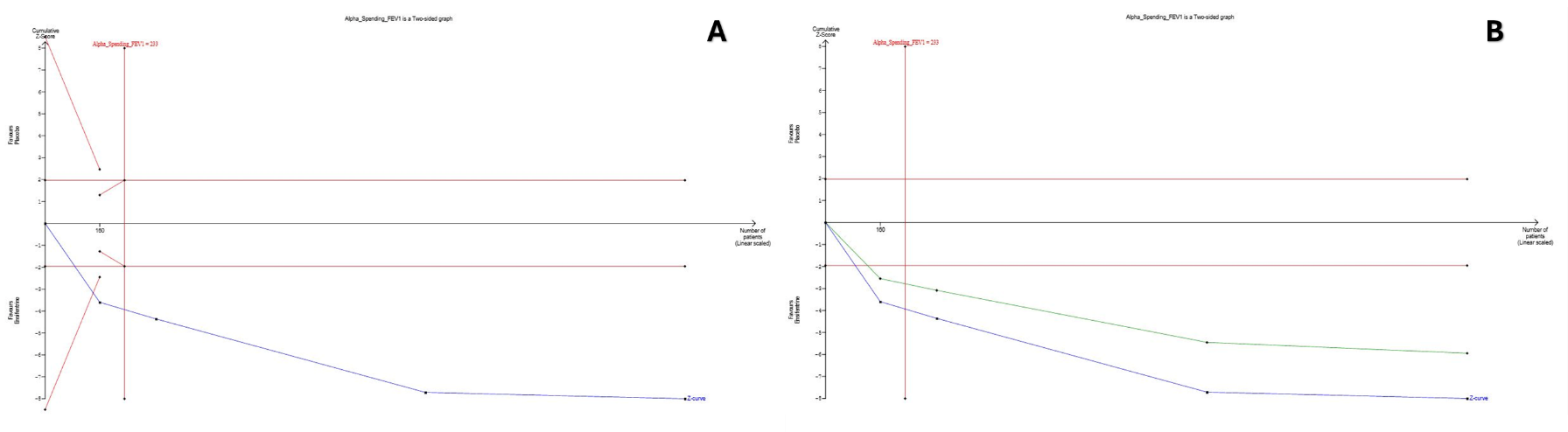


**(Supplementary figure. 3)** TSA on Mean Differences (MD) of change from baseline in average FEV1 (0–12h) between Ensifentrine 3 mg and placebo.

A) Average FEV1 cumulative z-curve passing the superiority boundary (True positive)

B) Average FEV1 penalized Z-curve passing the conventional boundary


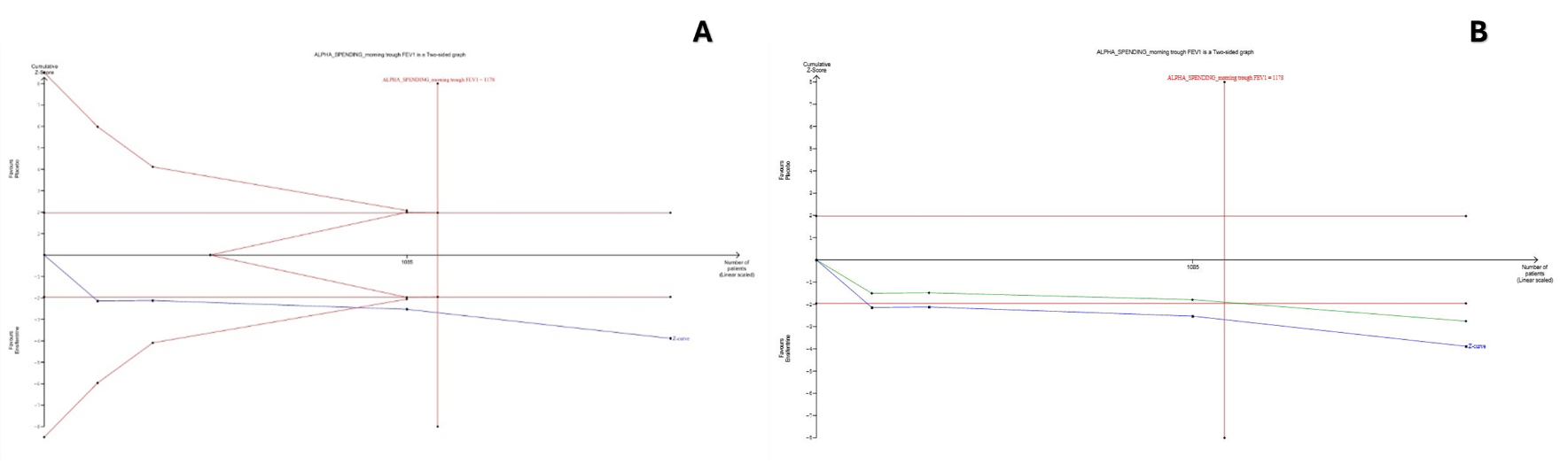


**(Supplementary figure. 4)** TSA on Mean Differences (MD) of Change from baseline in morning trough FEV1 between Ensifentrine 3 mg and placebo.

A) Morning trough FEV1 cumulative z-curve passing the superiority boundary (True positive)

B) Morning trough FEV1 penalized Z-curve passing the conventional boundary


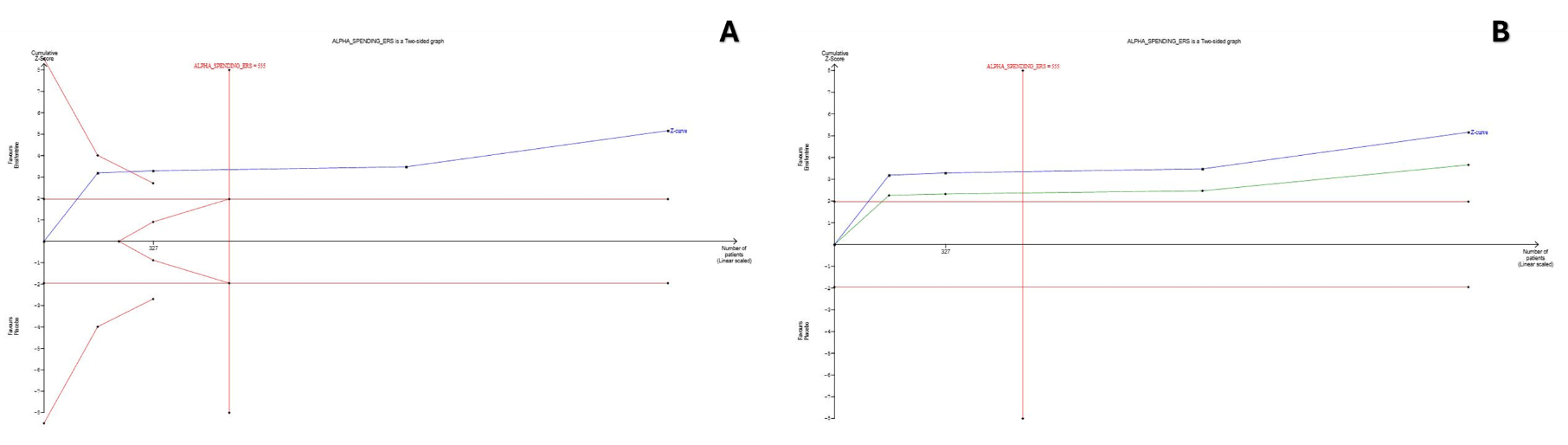


**(Supplementary figure. 5)** TSA on Mean Differences (MD) of Change from baseline in ERS between Ensifentrine 3 mg and placebo.

A) ERS cumulative z-curve passing the superiority boundary (True positive)

B) ERS penalized Z-curve passing the conventional boundary


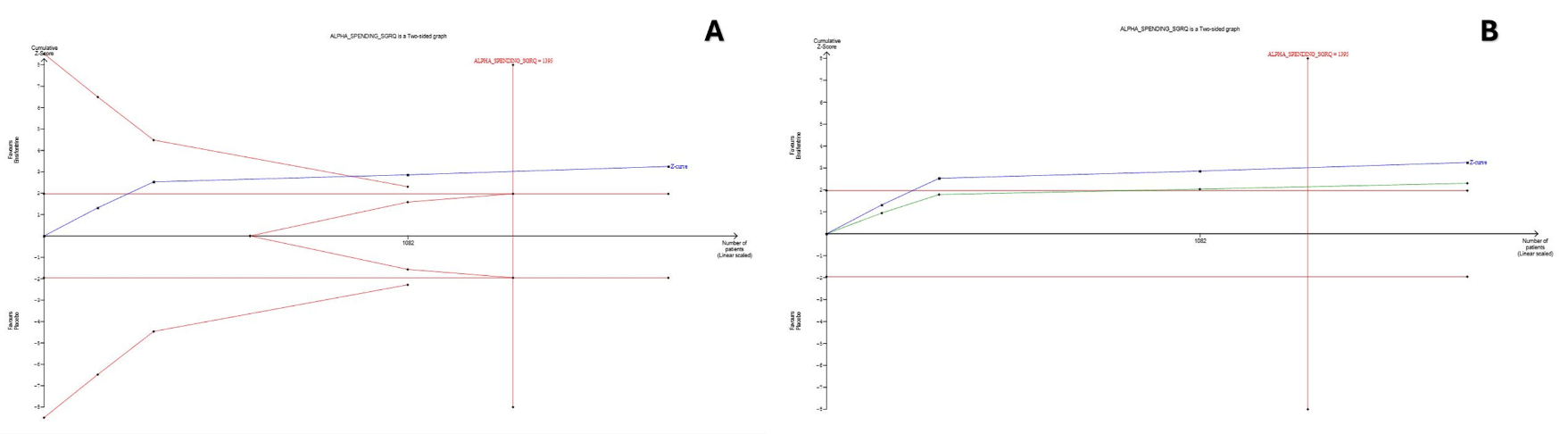


**(Supplementary figure. 6)** TSA on Mean Differences (MD) of Change from baseline in SGRQ between Ensifentrine 3 mg and placebo.

A) SGRQ cumulative z-curve passing the superiority boundary (True positive)

B) SGRQ penalized Z-curve passing the conventional boundary


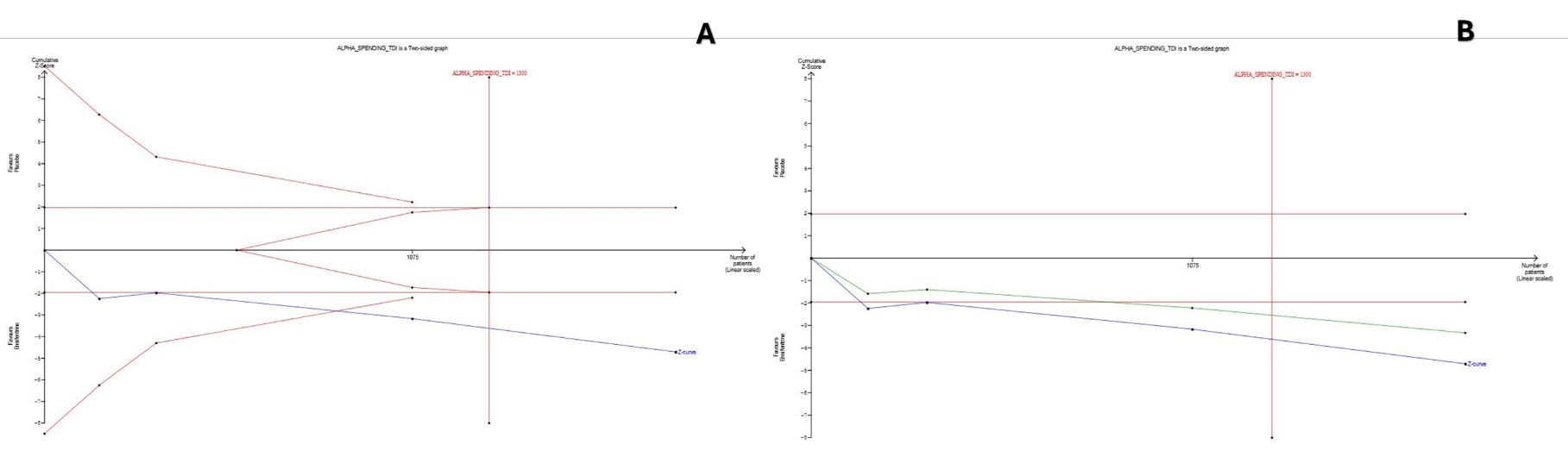


**(Supplementary figure. 7)** TSA on Mean Differences (MD) of Change from baseline in TDI between Ensifentrine 3 mg and placebo.

A) TDI cumulative z-curve passing the superiority boundary (True positive)

B) TDI penalized Z-curve passing the conventional boundary

**
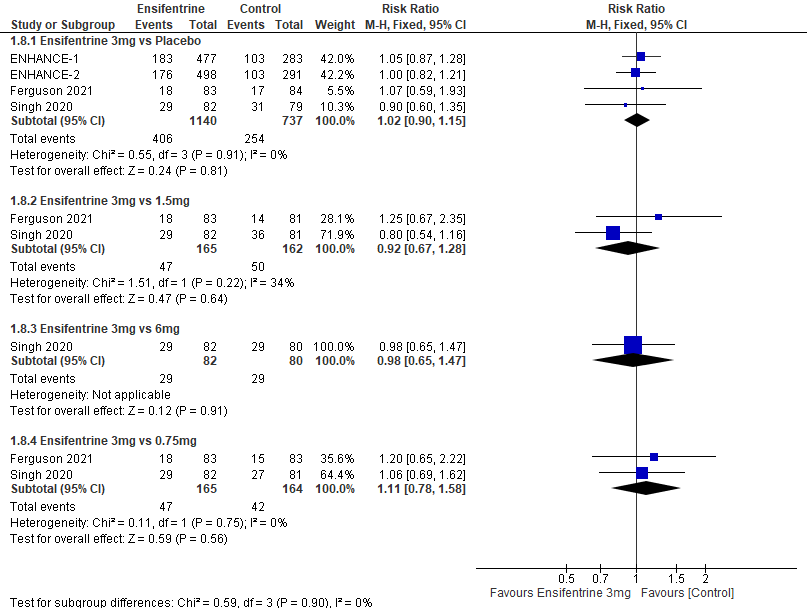
 (Supplementary figure. 8)** Forest plot comparing (RR) for TEAEs between Ensifentrine 3 mg and 0.75, 1.5 and 6 mg.

**
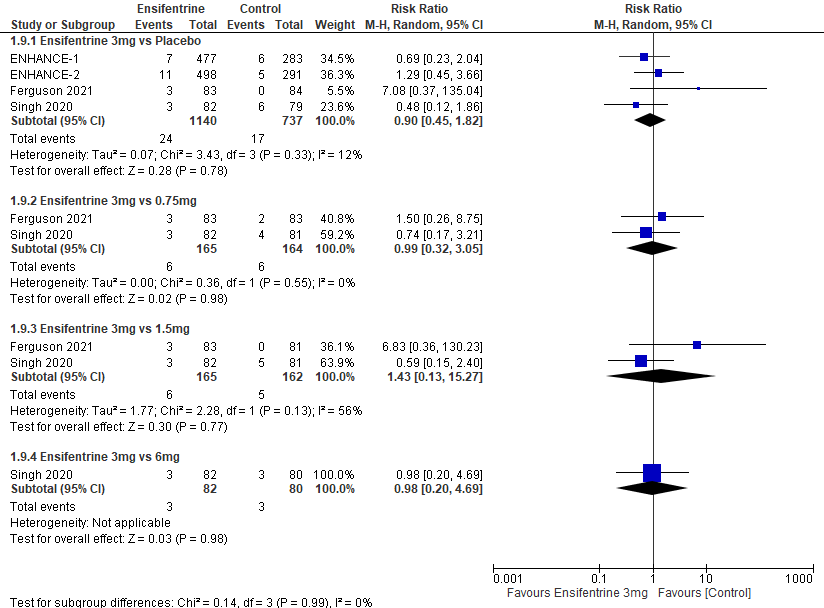
 (Supplementary figure. 9)** Forest plot comparing (RR) for COPD attacks between Ensifentrine 3 mg and 0.75, 1.5 and 6 mg.

**
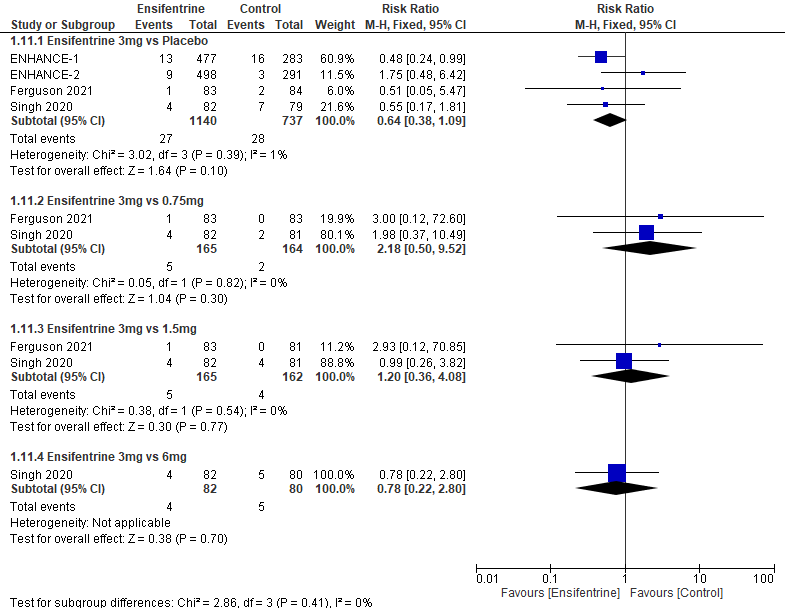
 (Supplementary figure. 10)** Forest plot comparing (RR) for Nasopharyngitis between Ensifentrine 3 mg and 0.75, 1.5 and 6 mg.


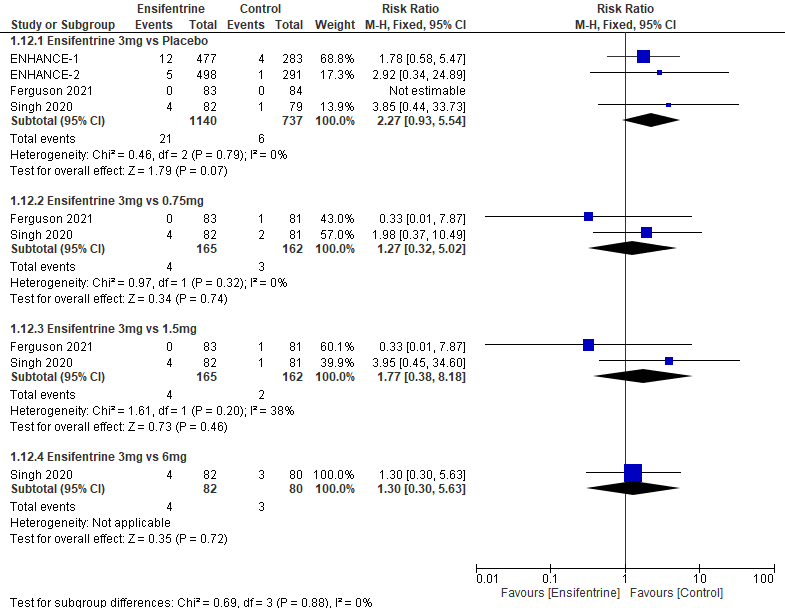


**(Supplementary figure. 11)** Forest plot comparing (RR) for Hypertension between Ensifentrine 3 mg and 0.75, 1.5 and 6 mg.


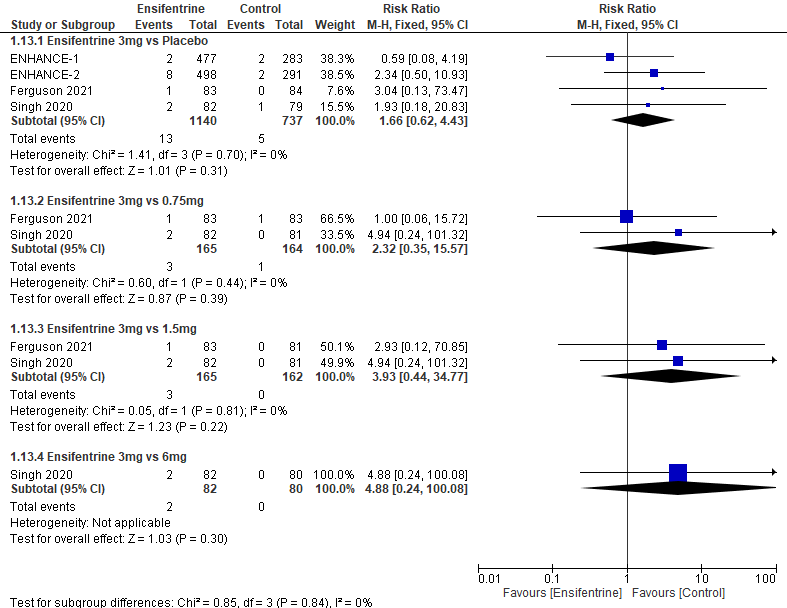


**(Supplementary figure. 12)** Forest plot comparing (RR) for Diarrhea between Ensifentrine 3 mg and 0.75, 1.5 and 6 mg.


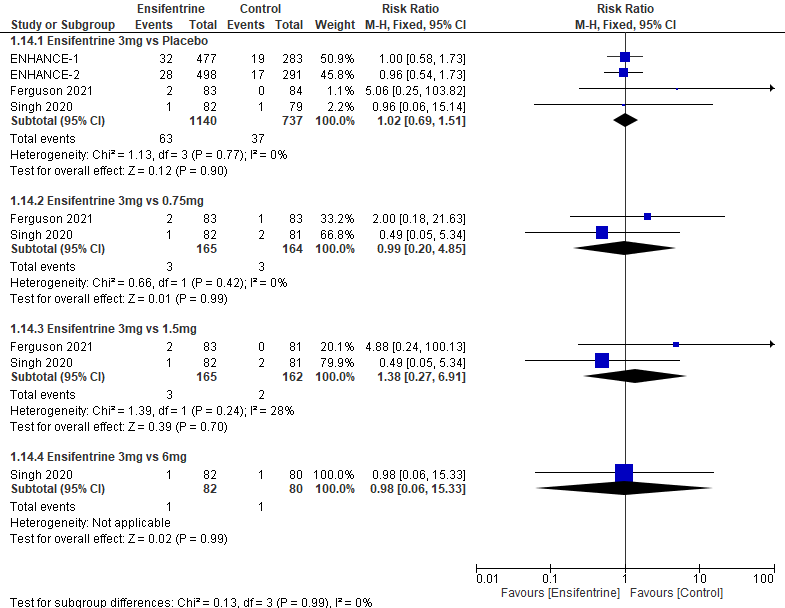


**(Supplementary figure. 13)** Forest plot comparing (RR) for Serious TEAEs between Ensifentrine 3 mg and 0.75, 1.5 and 6 mg.


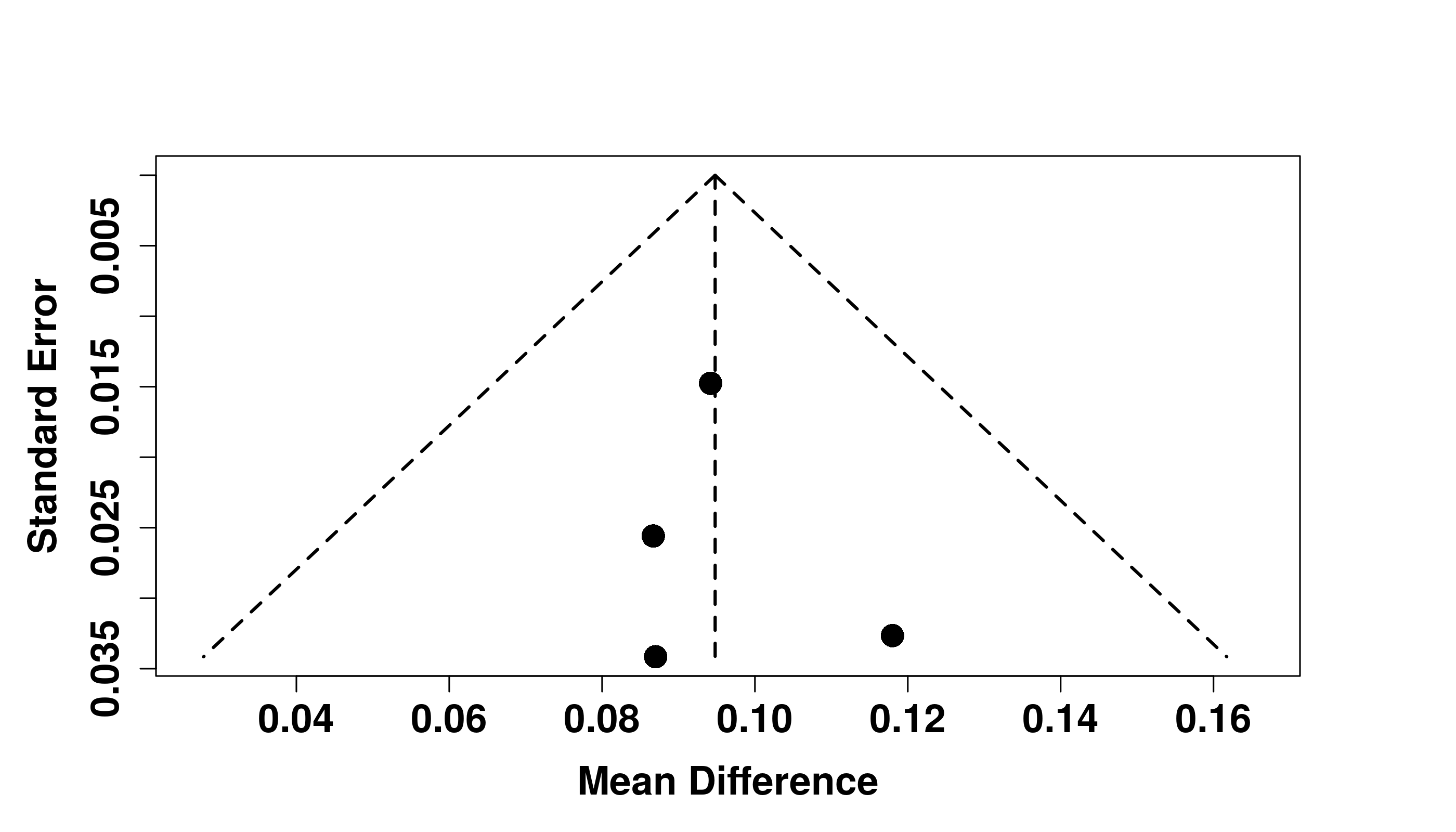


**(Supplementary figure. 14)** Funnel Plot for change from baseline in average FEV1 (0–12h) between Ensifentrine 3 mg and placebo.


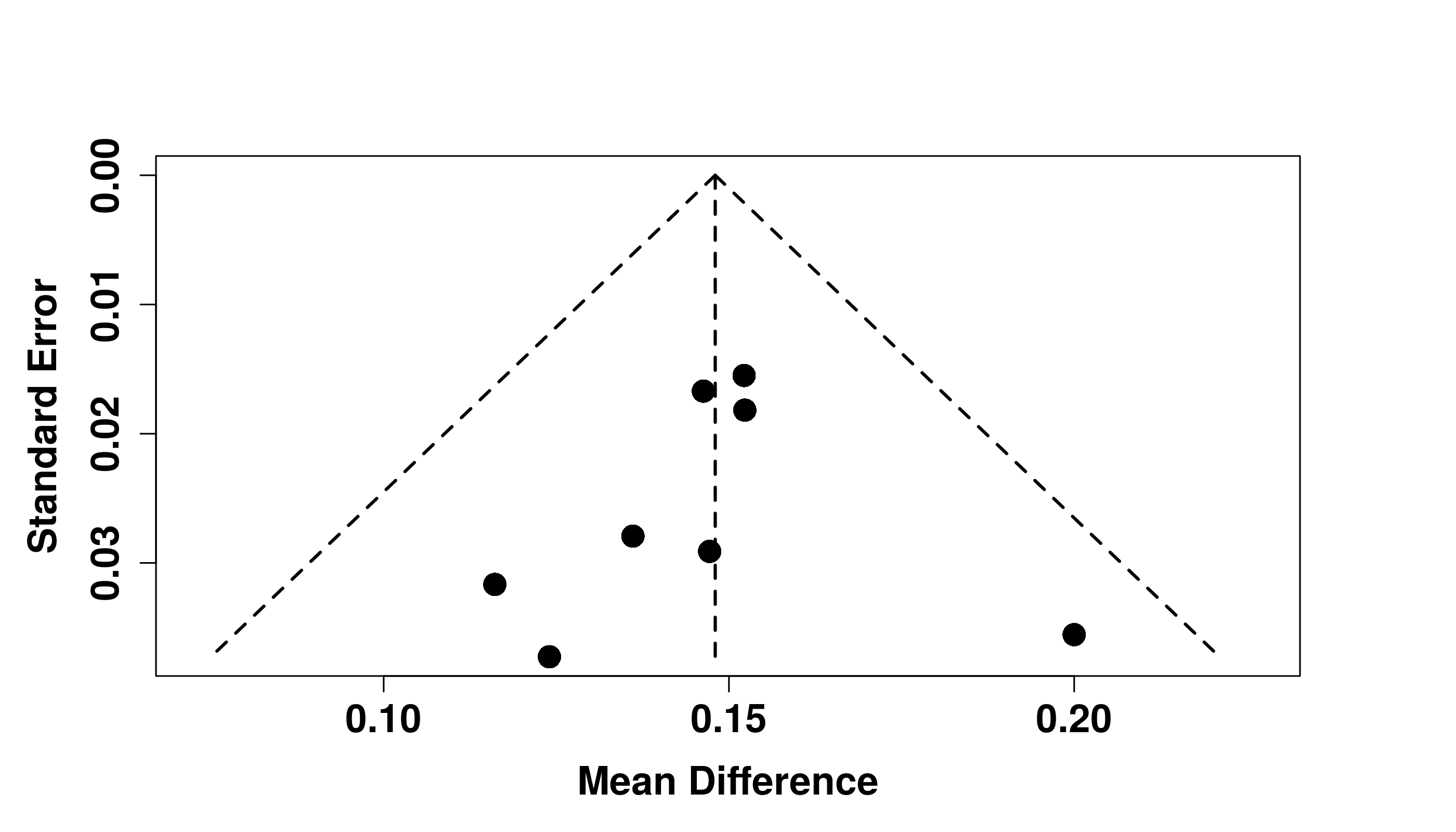


**(Supplementary figure. 15)** Funnel plot comparing (MD) for change from baseline in peak FEV1 (0–3h) Ensifentrine 3 mg and placebo.


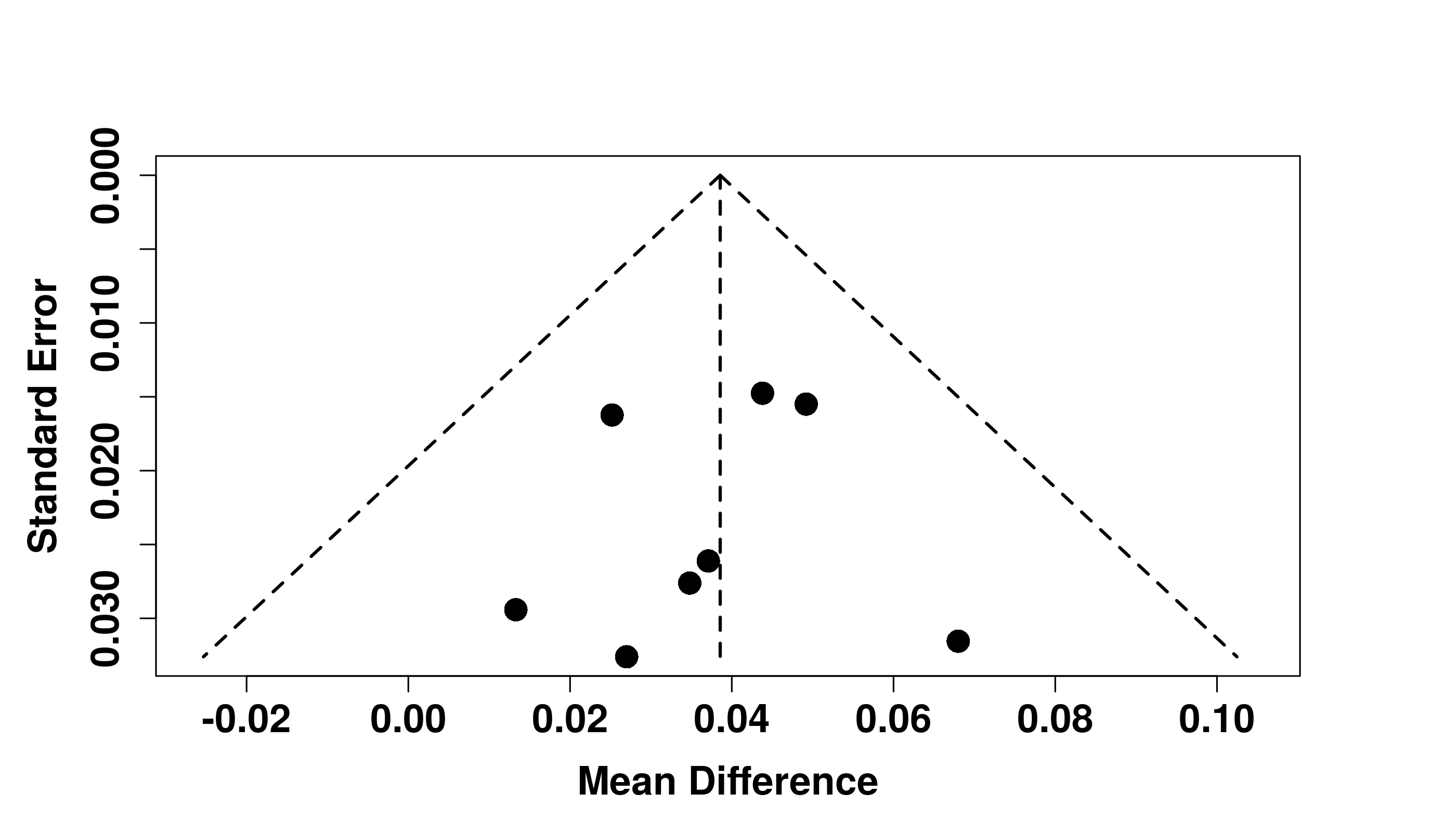


**(Supplementary figure. 16)** Funnel plot comparing (MD) for change from baseline in morning trough FEV1 Ensifentrine 3 mg and placebo.


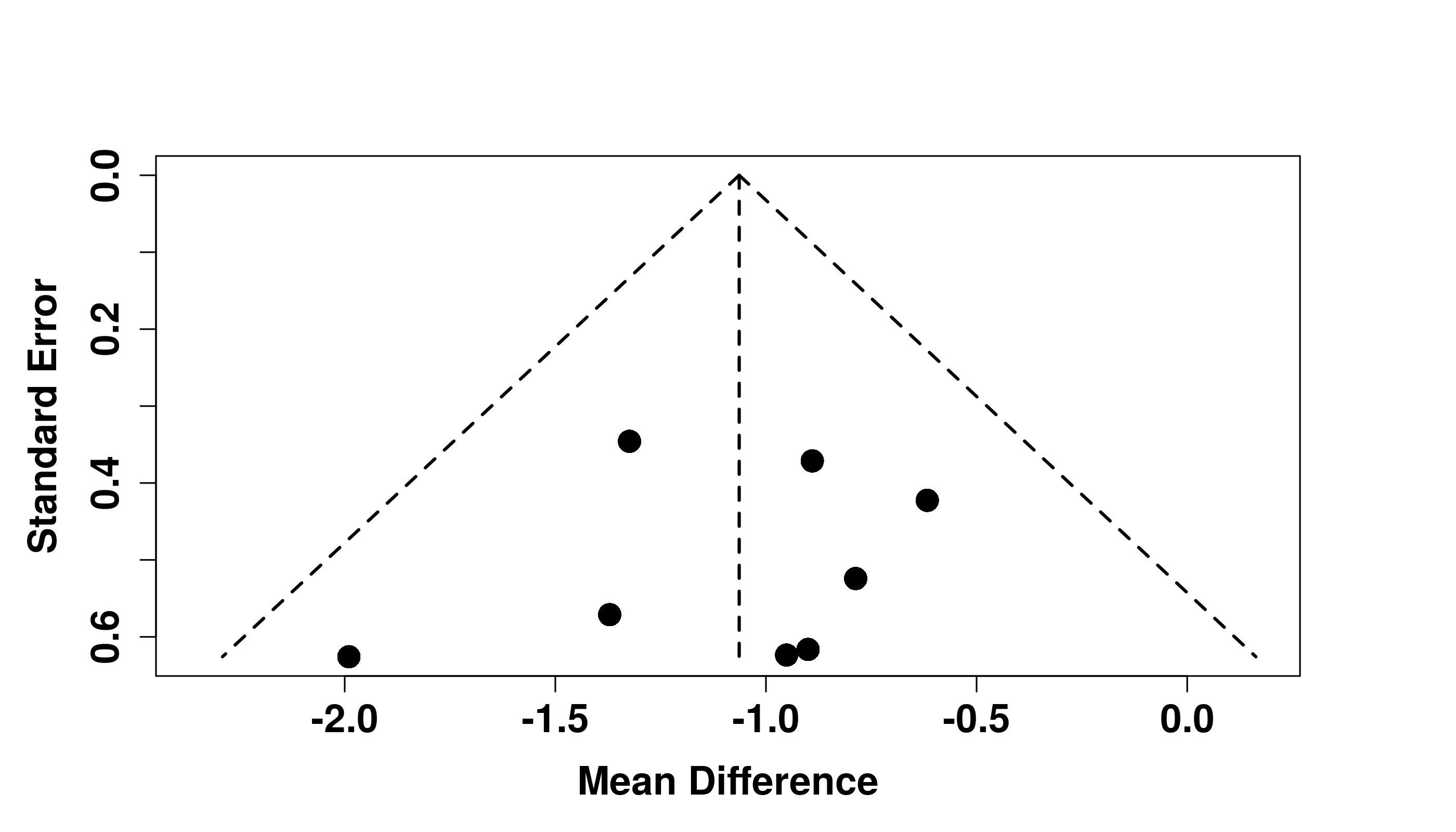


**(Supplementary figure. 17)** Funnel plot for change from baseline in ERS between Ensifentrine 3 mg and placebo.


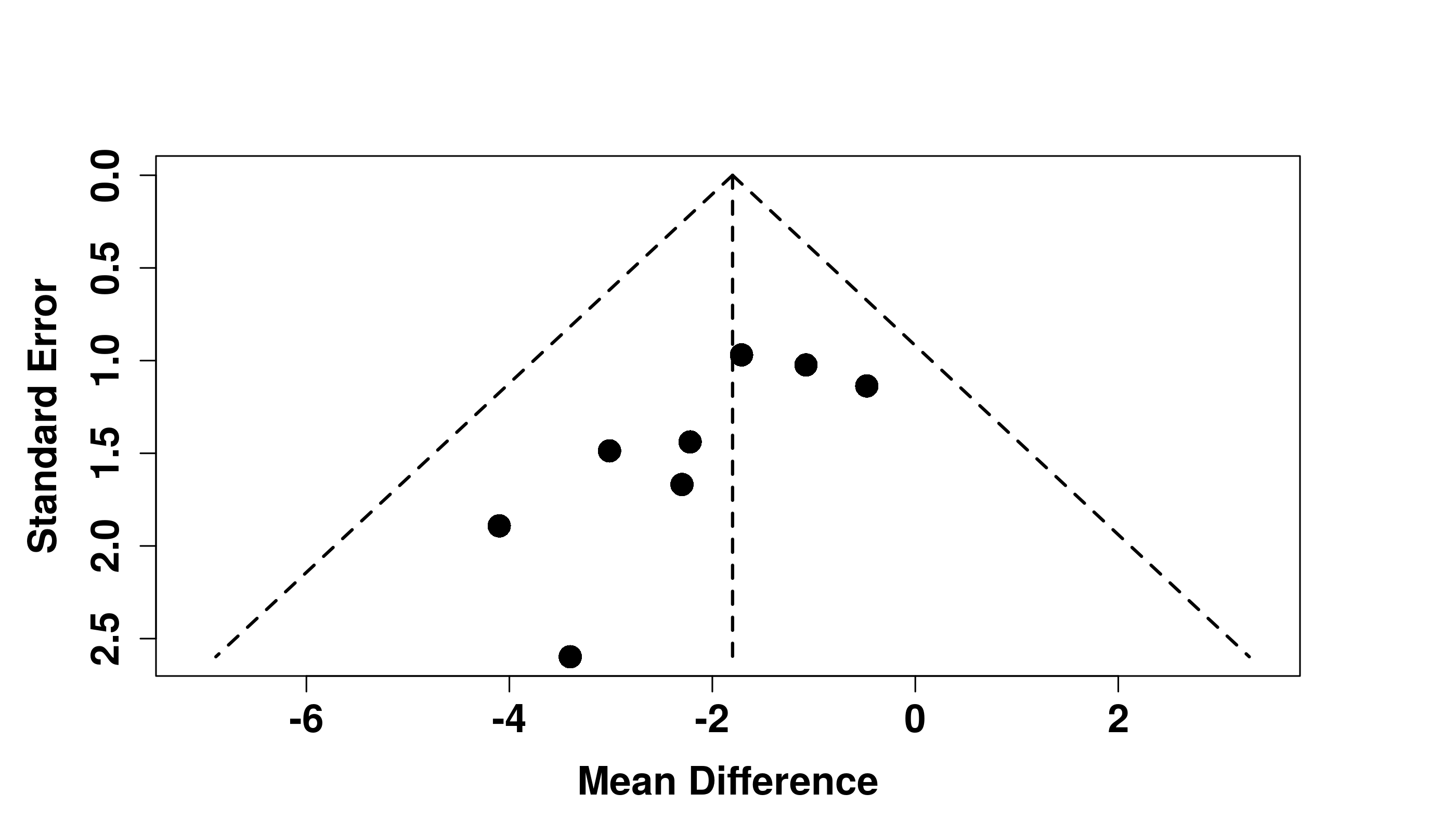


**(Supplementary figure. 18)** Funnel plot for Change from baseline in SGRQ between Ensifentrine 3 mg and placebo.


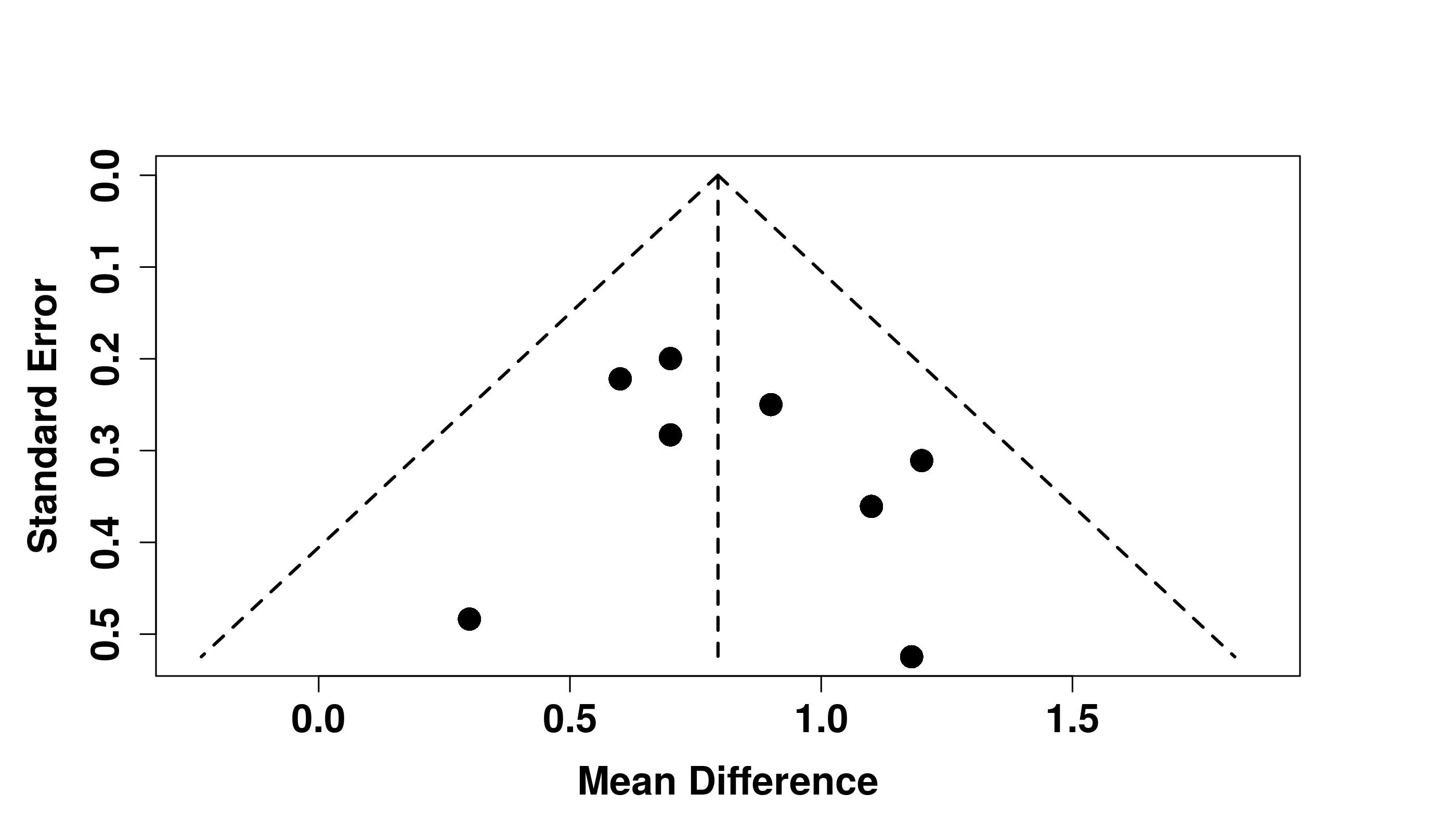


**(Supplementary figure. 19)** Funnel plot for Change from baseline in TDI between Ensifentrine 3 mg and placebo

**Supplementary Table S1. Comprehensive Search Strategy Used Across Databases**

| **Databases Searched** | **Search Strategy (Keywords and Boolean Operators)** |
| --- | --- |
| PubMed, Web of Science, Scopus | ("ensifentrine" OR "RPL-554" OR "Phosphodiesterase 3 and 4 Inhibitor" OR "Ohtuvayre") AND ("Chronic Obstructive Pulmonary Disease" OR "COPD"); Search included MeSH terms (where applicable), title/abstract/keyword fields. No language or date restrictions were applied during the initial search. |
